# A density functional study on the reactivity enhancement induced by gold in IrAu nanoalloys†

**DOI:** 10.1039/c7ra13347b

**Published:** 2018-03-14

**Authors:** Paula S. Cappellari, Germán J. Soldano, Marcelo M. Mariscal

**Affiliations:** INFIQC, CONICET, Departamento de Qumíca Teórica y Computacional, Facultad de Ciencias Químicas, Universidad Nacional de Córdoba (XUA5000) Córdoba Argentina marcelo.mariscal@unc.edu.ar pcappellari@exa.unrc.edu.ar

## Abstract

IrAu nanoalloys have been proven to have remarkable reactivity for several reactions. In this work, mixed IrAu nanoalloys of 8, 27, 48 and 64 total atoms were studied in different atomic compositions (Ir_*m*_Au_*n*_) using Density Functional Theory (DFT). A notable segregation tendency is observed, where Ir atoms are located in the inner part and Au atoms in the outermost region of the nanostructure. We found that IrAu nanoalloys present a distinctive synergistic effect with respect to reactivity. In addition, the projected density of electronic states (PDOS) energies were analyzed by examining the d-band shift to estimate the reactivity of various IrAu nanoalloys. Furthermore, the adsorption energies for the CO molecule in the domains of the Ir–Au interface were evaluated. In this sense, the addition of Au atoms to Ir clusters increases the reactivity of Ir by generating unoccupied orbitals near the Fermi level as indicated by the PDOS study.

## Abstract 2

The effects of chemical ordering on the stability and chemical reactivity on IrAu NAs of different sizes (8, 27, 48 and 64 total atoms) and compositions are studied using DFT calculations.

## Graphical abstract

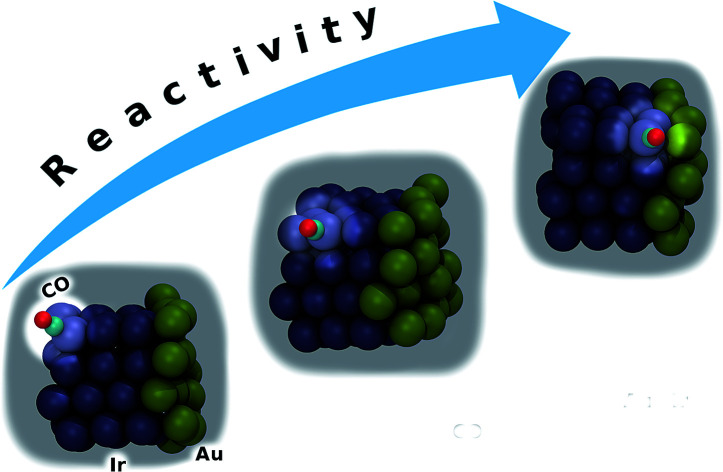

## 1 Introduction

Metal nanoparticles (NPs) are innovative materials with diverse and novel properties compared to their bulk counterparts.^1,2^ The essential characteristics of NPs, and in particular bimetallic nanoparticles (BNPs) or nanoalloys (NAs), depend on the method and the conditions in which they are synthesized. An improvement in their properties is generally observed due to synergistic effects upon alloying. The synergy between both metals, modulated by the different NA structural arrangements, introduces large diversity in reactivity, mainly due to the electronic tuning of metals.^3,4^

The morphology of a NA is specified when both its geometric structure and its chemical ordering pattern are specified.^5^ The chemical ordering pattern is the way in which the two elements are arranged within the geometric structure. Besides the size, shape and chemical composition, the spatial distribution and the stability of the elements constitute an excellent opportunity for the rational design of NAs.^6,7^ In fact, both the geometric shape and the energetic stability may drastically change with size.^8^

The great variety of compositions, structures, and features of metallic alloys has led to a wide range of new applications in electronics, engineering, and catalysis.^9–12^ With regard to the area of catalysis, several bimetallic systems have been reported and studied in the last few years.^13–16^ Bimetallic clusters offer fascinating prospects for the design of new catalysts. NA catalysts containing Pt with Ir or Re have found extensive use in the reforming of petrochemicals.^17^ Experimental reports show that the addition of the second metal improved the gold catalytic activity.^18,19^ On the other hand, gold can influence the activity and selectivity of some metals, such as palladium, platinum and ruthenium. Gold-containing bimetallic catalysts have found important industrial applications and represent an active research area. For the particular case of Pd and Pt, it was reported that the presence of gold can modulate the catalytic properties of H_2_ dissociation.^20^

Nilekar *et al.*^21^ have described that the presence of Ir and other transition metals in Pt NPs improves the preferential CO oxidation in hydrogen dramatically. Specifically regarding the IrAu system, it was shown that the addition of Au enhanced the chemisorptive and catalytic properties of bimetallic Ir–Au/γ-Al_2_O_3_ catalysts compared to the pure iridium samples in methylcyclopentane (MCP) hydrogenolysis.^22^ It has also been reported that IrAu NAs displayed enhanced activity in ethanol oxidation to acetaldehyde, outperforming their monometallic counterparts.^18^ In this respect, the effects of gold addition to iridium catalysts for citral hydrogenation over Ir–Au/TiO_2_ catalysts were investigated by Rojas *et al.*^23^ Additionally, the improvement of the catalytic activity for the oxidation of CO of Ir–Au/rutile catalysts, which could be due to a synergetic effect caused by the combination of gold and iridium in small particles, has been reported by Bokhimi *et al.*^24^

Despite the experimental evidence on the synergic effect between iridium and gold in nanoalloys, a systematic first principles study of such systems and in addition, a fundamental understanding of the physical picture behind this phenomenon, is still missing. Jiménez-Díaz *et al.*^25^ studied the reactivity of CO and O_2_ on 20-atom IrAu NAs by DFT. The nanoclusters on which the adsorption was tested were relaxed using the Gupta potential; consequently, the metal atoms are highly coordinated. In contrast, DFT studies suggest that such clusters are characterized by low coordinated atoms forming cubic building blocks.^26–29^ Clearly, a new approach on more realistic clusters is needed.

Theory can also shed light into an experimental blindspot, which is the spatial distribution of species. Previous works^25,29,30^ have attempted to do so, although the cluster sizes investigated were always smaller than the experimental ones (1–2 nm).^15,23,24^ Therefore, the present study represents the first theoretical approximation to “real” synthesized nanoalloys. In this report, IrAu NAs of different sizes (8, 27, 48 and 64 total atoms) and compositions are studied using DFT calculations in order to understand the effects of chemical ordering on the stability and chemical reactivity of these nanostructures. The latter was evaluated using CO as a probe molecule, comparing its adsorption energy on IrAu NAs and the corresponding pure metal clusters. The DFT results presented here reveal an improvement in the reactivity of Ir by Au atoms already evidenced experimentally and subsequently, by means of the study of PDOS, the reason for such improvement could be understood.

## 2 Methodology

### 2.1 Generation of nanostructures

The number of local minima in the Potential Energy Surface (PES) increases exponentially (*i.e.* e^*N*^). Consequently, DFT methods to obtain total minima configurations become increasingly costly in computational terms. For this reason, several global optimization methods combine approaches in which the most stable candidates are first selected by means of semiempirical potentials to later be refined with high precision DFT. Pure Au clusters were generated using this type of method, based on the “big bang” algorithm.^31^ 50 000 clusters with random atomic positions were the starting point of the simulated annealing up to 400 K for 10 ps using the Embedded Atom Method^32^ (EAM). The resulting 40 most stable structures were relaxed by DFT with low accuracy. Finally, the resulting 20 most stable were relaxed with high accuracy DFT. Unfortunately, to date, there is no reliable potential that can accurately describe both the Ir–Ir and the Ir–Au interaction, so the above algorithm cannot be used for our purposes. Still, our aim is not to find the lowest energy configuration of these nanostructures, but to study the effect of gold on the geometry and reactivity of Ir clusters. The construction of Ir clusters was guided by the combination of building blocks found for Ir clusters that had between 10 and 20 atoms.^27,30,33^ Evidence of these building blocks has also been observed in STEM (Scanning Transmission Electron Microscopy) images.^24,34^ IrAu NAs were constructed as cubic structures and also guided by the structure of the most stable Ir clusters. Ir atoms were progressively replaced by Au atoms at several positions. All these structures were relaxed using DFT.

### 2.2 DFT

Density Functional Theory (DFT) calculations were performed using the Quantum Espresso/PWSCF code.^35^ Vanderbilt ultrasoft pseudopotentials^36^ were used with the Perdew–Burke–Ernzerhof (PBE)^37^ approximation. For low precision DFT calculations, a 38 Ry kinetic energy cutoff and a 380 Ry charge density cutoff were used. For high precision DFT these values are 50 Ry and 500 Ry, respectively. The reciprocal space was sampled with the gamma point approximation using the Monkhorst–Pack method.^38^ All these parameters were carefully optimized. A Gaussian broadening of 0.01 Ry was applied. Relaxations were converged when the forces were less than 0.02 eV Å^−1^. Unit cell dimensions were such that the clusters were separated by at least 12 Å from their periodic images. Due to the natural magnetic properties of iridium atoms, spin polarizations were considered both for the pure Ir clusters and for the IrAu NAs.

### 2.3 Energetics

In order to analyze cluster stability, some energetic quantities need to be defined. The formation energy per atom was calculated according to:1
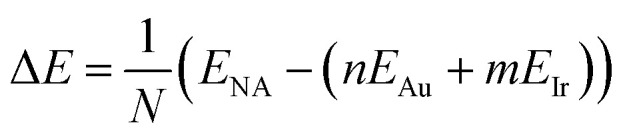
where *N* is the total number of atoms (*N* = *n* + *m*), *n* is the number of gold atoms and *m* is the number of iridium atoms, *E*_Ir_ is the total energy of a spin-polarised atom Ir cluster and *E*_Au_ is the energy of an isolated Au atom. *E*_NA_ is the total energy of an *N*-atom IrAu NA.

The energy of chemisorption of a CO molecule onto a metal cluster is defined as2
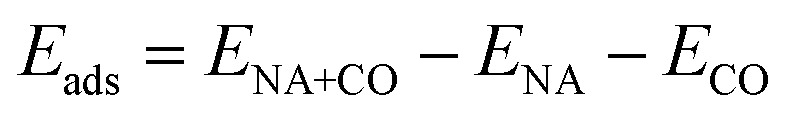
where *E*_NA+CO_, *E*_NA_ and *E*_CO_ are the energies of the nanoalloy with CO, the bare nanoalloy, and the isolated CO molecule, respectively. With this definition, a more negative *E*_ads_ value represents a more strongly bound CO molecule.

The relative CO adsorption energy is defined as the difference between the adsorption energy on pure Ir and that on the IrAu NA at analogous sites.3
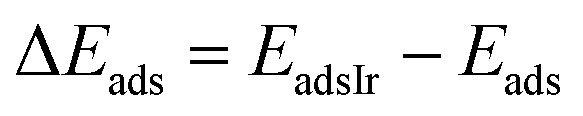


## 3 Results

### 3.1 IrAu NA structures

**Fig. 1:**
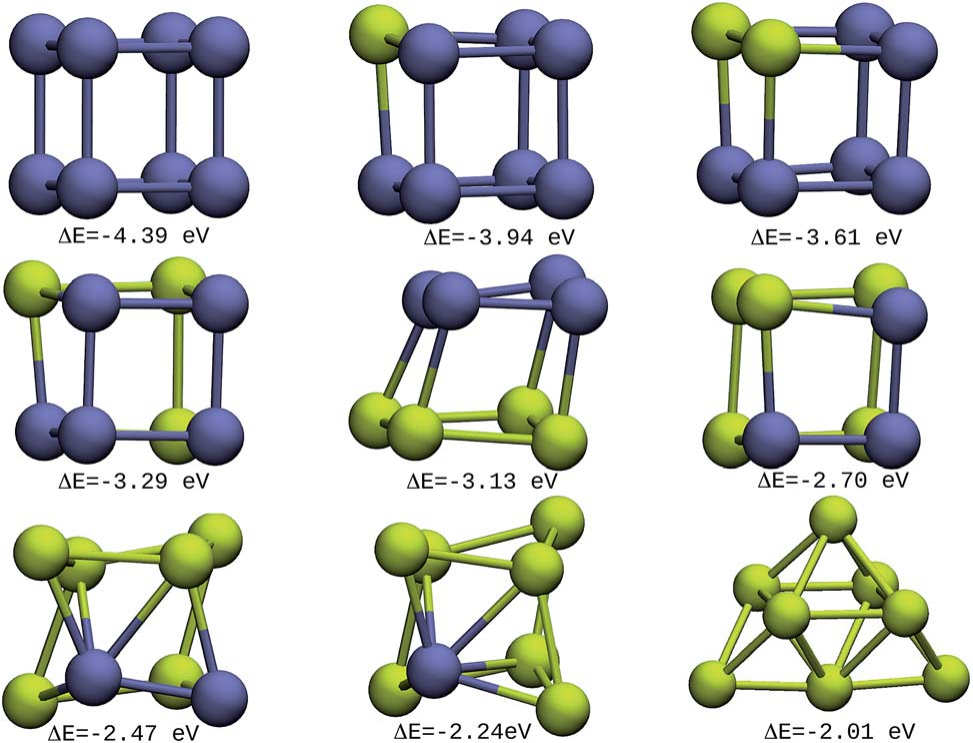
8-atom IrAu NAs. The green spheres represent atoms of Au and the blue spheres atoms of Ir. Under each structure the formation energy Δ*E* is reported.

The resulting most stable 8-atom IrAu NA structures are exhibited in Fig. 1. The structures we found for pure 8-atom Au and Ir are 3-dimensional clusters, similar to those found in previous theoretical works.^30,33,39–43^ A clear trend in 8-atom IrAu NAs is that while Au atoms lose the crystallographic arrangement (with respect to the Ir crystal structure), the Ir atoms conserve it.

It is important to mention that gold and iridium are impossible to distinguish from one another using the Z-contrast annular dark-field images in HAADF-STEM (High-Angle Annular Dark-Field Scanning Transmission Electron Microscopy) due to the proximity of their atomic number (77 and 79, respectively).^24^ In this regard, the study by means of DFT of IrAu NAs with high atomic numbers would generate a complete approach for the experimental field as well as assist in interpreting the different environments of Ir and Au in STEM images. The high energy of the more mixed clusters (not shown) reveals clear segregation phenomena among the two metals, as described in a study on first principles calculations of transition metal binary bulk alloys by Aspera *et al.*^44^

In addition, it is found that Ir atoms are more unstable at the undercoordinated sites than Au. This effect causes Au atoms to occupy locations on the periphery of the nanostructure, whereas Ir atoms tend to locate in the innermost region. This last trend was clearly recorded in the IrAu structures with *N* = 8, as well as 2D nanostructures with *N* = 9 (see Fig. S1, ESI†). This phenomenon has been reported for different metals.^7,45,46^ In particular, this type of performance has been recorded in IrAu NA structures previously by Davis *et al.*^29^ and Jiménez-Díaz *et al.*^25^

**Fig. 2:**
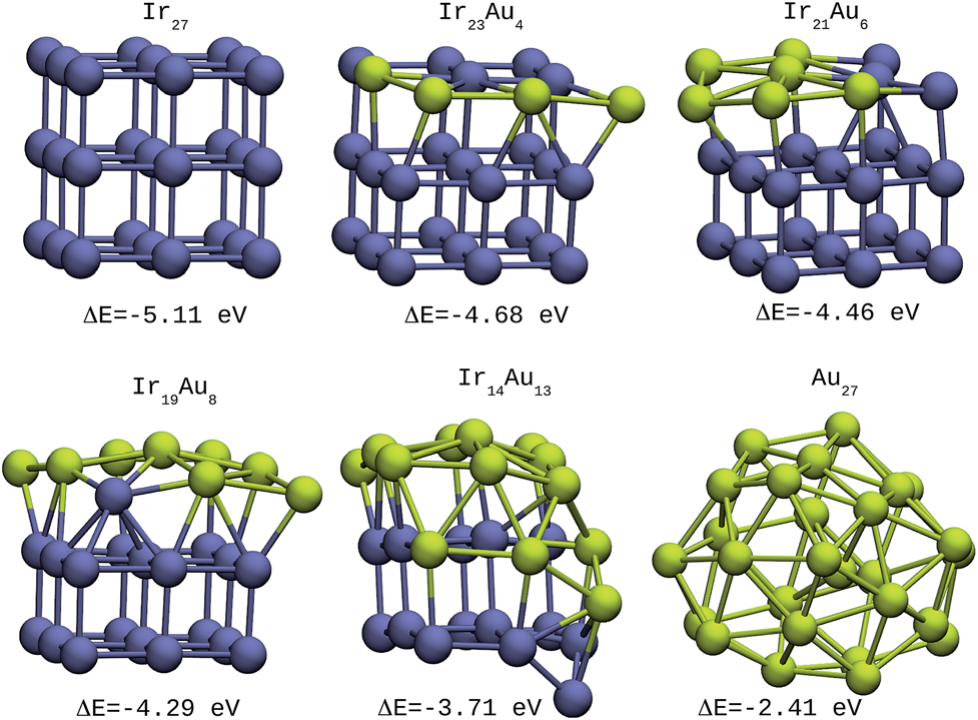
27-atom IrAu NAs. The green spheres represent atoms of Au and the blue spheres atoms of Ir. Under each structure the formation energy Δ*E* is reported.

**Fig. 3:**
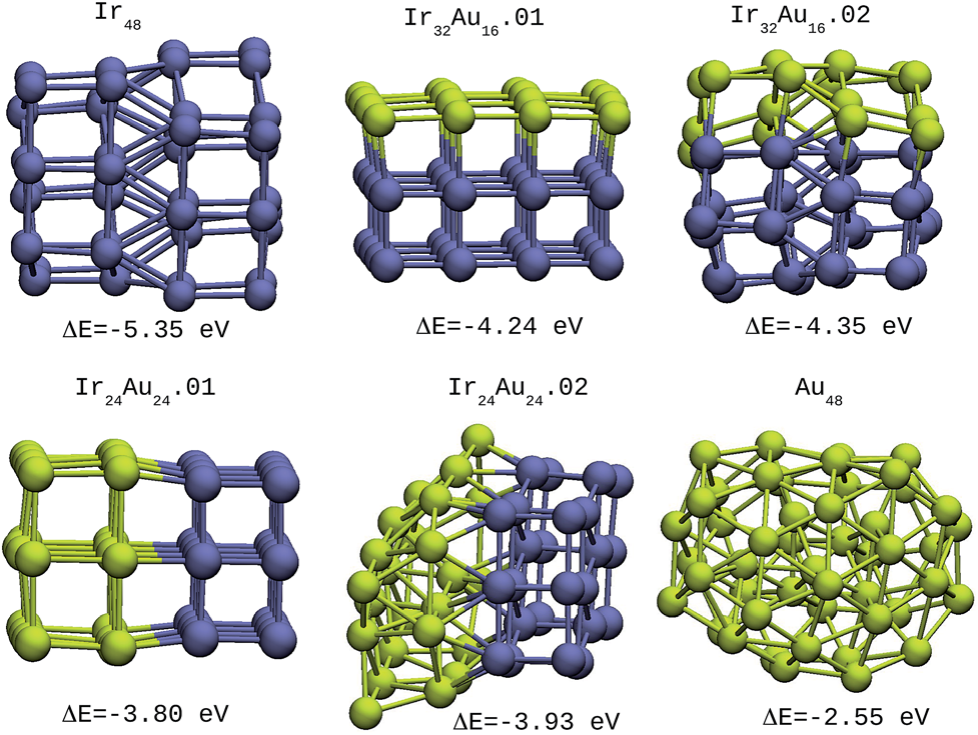
48-atom IrAu NAs. The green spheres represent atoms of Au and the blue spheres atoms of Ir. Under each structure the formation energy Δ*E* is reported.

**Fig. 4:**
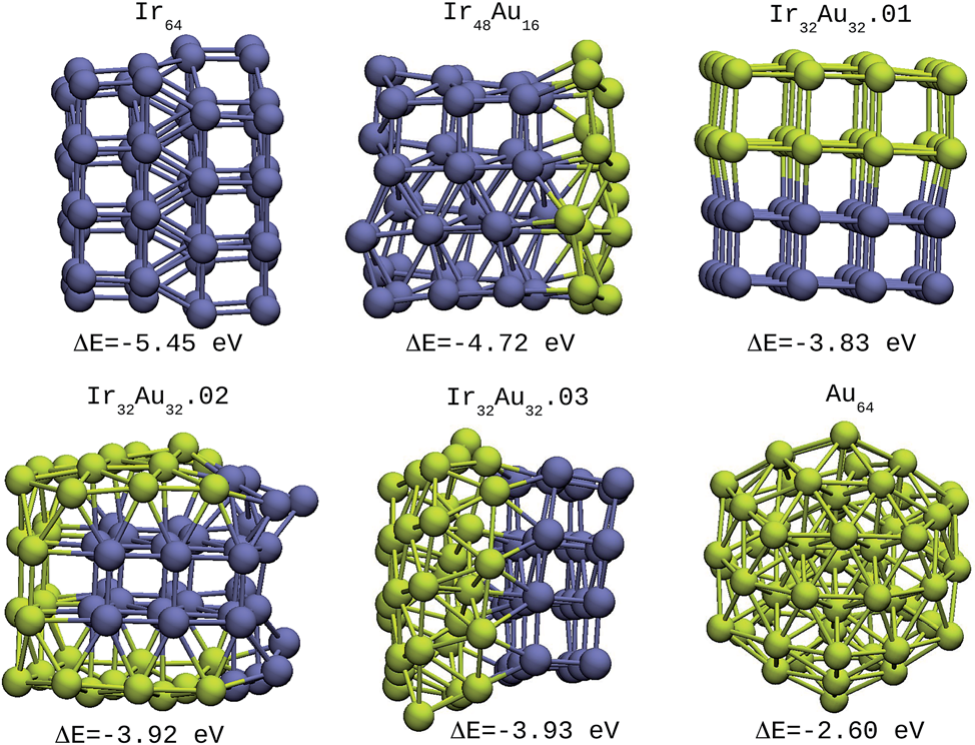
64-atom IrAu NAs. The green spheres represent atoms of Au and the blue spheres atoms of Ir. Under each structure the formation energy Δ*E* is reported.

The same trends were found for larger clusters, as shown in Fig. 2–4. In the case of 27-atom IrAu NAs, four different isomers were tested for each type of atomic distribution. The formation energy differences between them oscillate in the range of 0.1–0.2 eV (see Fig. S2 ESI†). Fig. 2 shows the most stable isomers found here for mixtures of different atomic compositions for clusters of 27 atoms.

For the IrAu NAs with *N* = 48 and 64 two possible compositions for each atomic distribution were studied, which are shown in Fig. 3 and 4. Isomers containing “house” blocks were found to be more stable than those with cubic shapes. If the formation energy of Ir_32_Au_16_.01 is compared with that of Ir_32_Au_16_.02 (Fig. 3) there is a stabilization of 0.11 eV in the latter. The repetition of “house” blocks is clearly perceived for the insertion of Ir atoms in Ir_32_Au_16_.02, while the Au environment remains amorphous. The spin multiplicity is in agreement with what has been described by Davis *et al.*,^33^ where mixed structures are analyzed starting from Ir cubes. In cases where the structure is maintained, the spin multiplicity is low (1–3), similar to the trend seen for IrAu NA, in Table 1.

**Table 1:** Formation energies (Δ*E*) and multiplicities (2*S* + 1) of the various IrAu NA compositions shown in Fig. 2–4

IrAu BNA	Δ*E*/eV	(2*S* + 1)
Ir_27_	−5.11	2
Ir_23_Au_4_	−4.68	2
Ir_21_Au_6_	−4.45	2
Ir_19_Au_8_	−4.29	2
Ir_14_Au_13_	−3.71	3
Ir_14_Au_13_	−3.71	3
Ir_48_	−5.35	3
Ir_32_Au_16_.01	−4.24	3
Ir_32_Au_16_.02	−4.35	2
Ir_24_Au_24_.01	−3.80	1
Ir_24_Au_24_.02	−3.93	2
Ir_64_	−5.45	2
Ir_48_Au_16_	−4.72	2
Ir_32_Au_32_.01	−3.83	2
Ir_32_Au_32_.02	−3.92	3
Ir_32_Au_32_.03	−3.93	2

We remark that the geometries are fully relaxed at 0 K as this is straightforward in DFT calculations, however it should be taken into account that in “real” experimental systems these materials are subjected to high temperatures, where a large number of isomers can “live” at the same time.

In order to evaluate the degree of crystallinity of these systems, the pair correlation functions *g*(*r*) were analyzed for the most relevant structures, shown in ESI Fig. S3.† This analysis confirms that Ir atoms present a high degree of crystal ordering while Au atoms are rather disordered. This result contributes to the hypothesis that the highly ordered and randomly distributed atoms in the TEM images correspond to the Ir and Au atoms, respectively.^24,34^

**Fig. 5:**
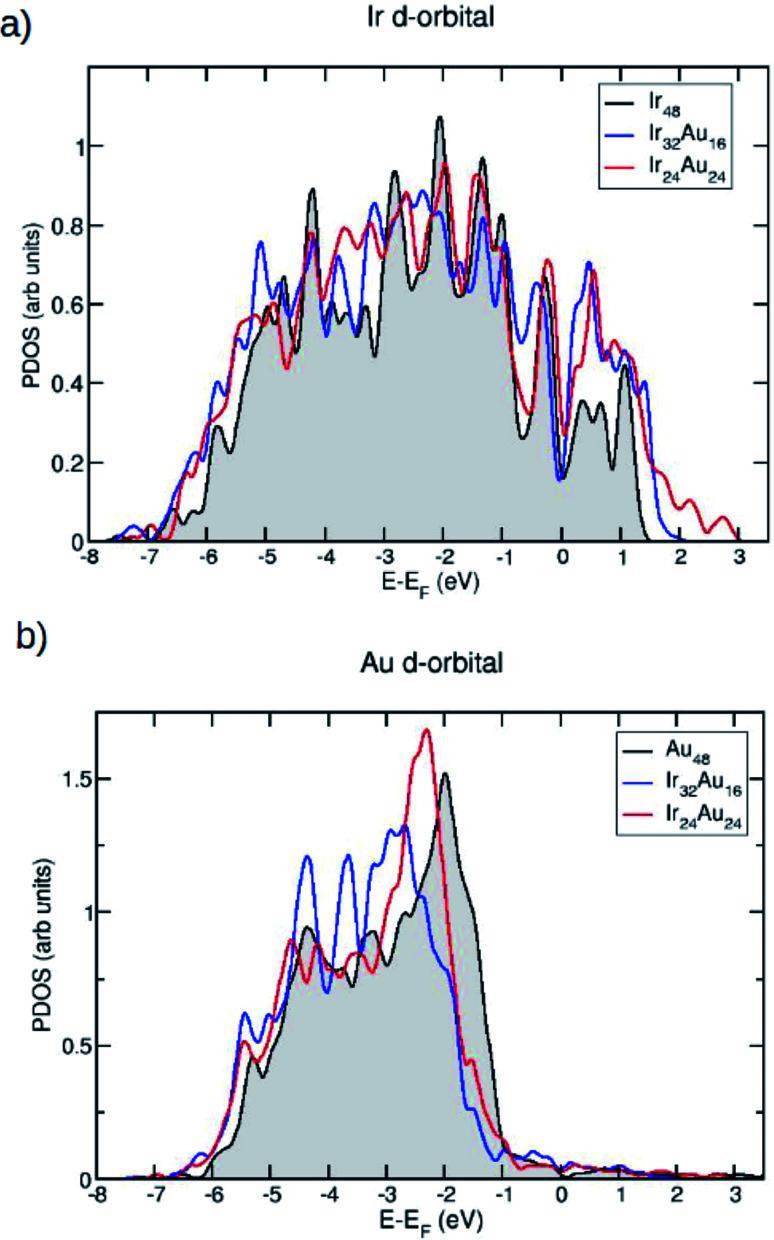
The projected density of electronic states (PDOS) plots of the d orbitals (a) of the average Ir atoms on different structures Ir_48_, Ir_32_Au_16_ and Ir_24_Au_24_, and (b) of the average Au atoms on different structures Au_48_, Ir_32_Au_16_ and Ir_24_Au_24_. The energy is relative to the energy of the Fermi level.

Since we are interested in the electronic properties and even more so in the reactivity of these IrAu NAs, the projected density of states (PDOS) for the most stable structures was calculated. In Fig. 5, the PDOS is shown for the case of (IrAu)_48_ with different compositions (other cases are shown in the ESI, Fig. S4†).

We focus our analysis on the d orbitals, which are known to actively participate in catalytic reactions.^47^ In fact, it has been shown that the reactivity of transition metals is directly related to the d-band shape, width, and position.^48,49^Fig. 5a and b show the PDOS of the d-band for the Ir and Au atoms in the mixture, respectively. In each case they are compared with the PDOS of the pure clusters. An upward shift of the Ir d-band center is revealed, as shown in Table 2.

**Table 2:** Values of the d-band center (in eV) for Ir atoms and Au atoms in the structures Ir_48_, Au_48_, Ir_32_Au_16_ and Ir_24_Au_24_ shown in Fig. 6

IrAu nanoalloy	Ir d-band center	Au d-band center
Ir_48_	−2.54	—
Au_48_	—	−3.14
Ir_32_Au_16_	−2.48	−3.21
Ir_24_Au_24_	−2.20	−3.12

In contrast, for Au atoms, a slight downward shift upon alloying is observed. These d-band center displacements induced by gold have also been observed for RhAu nanostructures.^50^ An upward displacement of the d-band center is usually associated with an increase of reactivity: the d-band tends to form bonding and antibonding states at energies below and above its original value, respectively. If the d-band of the pure metal is above the Fermi level, the upward shift induced by the other metal tends to empty these antibonding states, resulting in a stronger reactant–metal bond.

### 3.2 CO adsorption on IrAu NAs

**Fig. 6:**
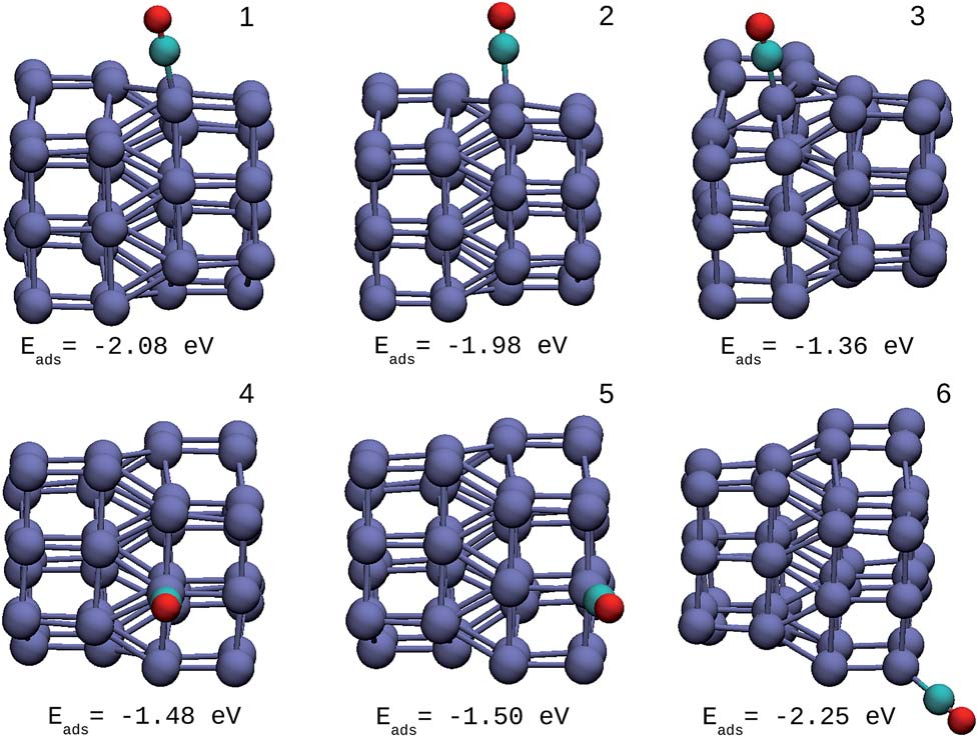
Different positions of molecular CO adsorbed on the most stable Ir_48_ clusters with CO adsorption energies. The numbers on each structure symbolize the different Ir atomic positions where CO is adsorbed.

The oxidation reaction of CO has been studied extensively for different types of nanocatalyst.^25,34,50^ This reaction is considered as a simple model to find catalytic properties that can be extrapolated to reactions of greater complexity. In addition, from an experimental viewpoint, the adsorption of CO in metal systems is used as a probe to test surface effects that are difficult to detect by other techniques. Fig. 6 shows the structures and adsorption energies of CO molecules adsorbed on the top configuration in different atomic positions for the Ir_48_ cluster. Several high-symmetry adsorption sites were tested. The on-top sites were found to be the most stable ones in accordance with previous results. The same was found for CO on the IrAu and Au clusters, in agreement with previous theoretical predictions.^24,25^ In order to take the different metal coordinations into account, six different on-top sites were inspected for CO adsorption on Ir_48_. It was found that the strength of CO adsorption is dependent on the coordination of Ir atoms, being stronger for low coordinated atoms (edges and vertexes) and weaker for higher coordinations (terraces and core atoms).

The adsorption energy on the terrace atoms in Ir_48_ is between 0.1 and 0.9 eV higher than that on the low coordinated atoms. CO adsorption on Au was also calculated and found to be at least 1 eV higher in energy than that on the Ir NPs. Other data to consider is that the average distance of Ir–C for all the adsorption sites and in the different structures was in the range of 1.88–1.90 Å, while the bond distance of CO under vacuum is 1.15 Å, and after the allocation of CO in the sites, this was maintained between values of 1.16 and 1.19 Å.

The same procedure was applied for the other IrAu NAs (*N* = 8, 27, and 64). In all the cases studied, the same behavior for the adsorption of the CO molecule was found. The adsorption of CO in the pure Au clusters was investigated. CO adsorbs more strongly on Ir_48_ than Au_48_, with adsorption energies of −2.25 eV and −1.03 eV, respectively, for the best atomic adsorption site (see Fig. S5 in the ESI†).

**Fig. 7:**
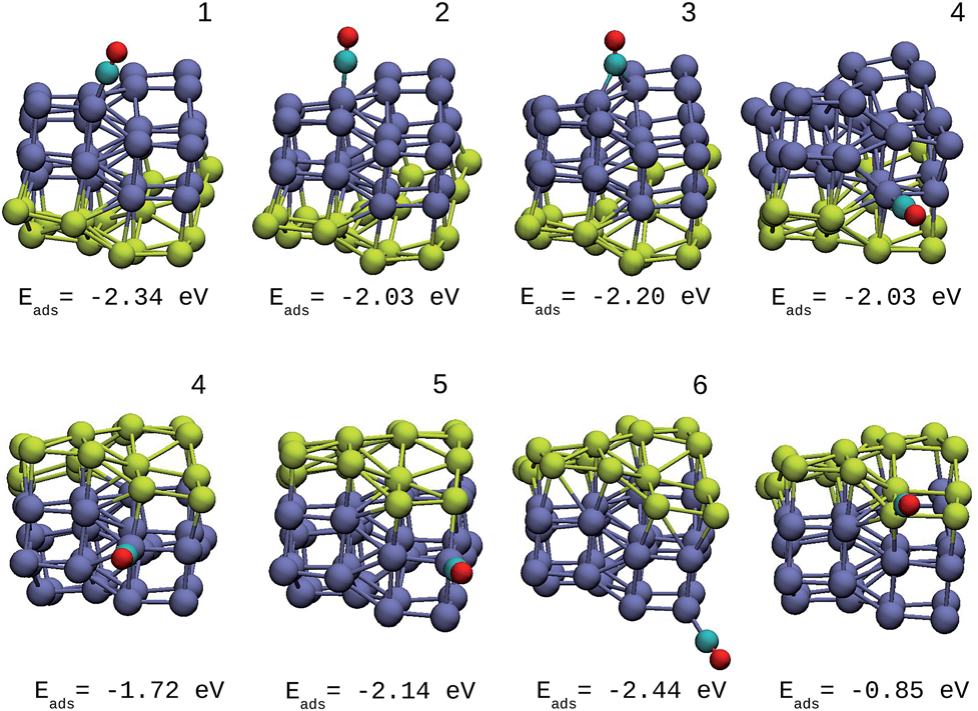
Different positions of molecular CO adsorbed on the most stable Ir_32_Au_16_ NA with CO adsorption energies. The numbers on each structure symbolize the different Ir atomic positions where CO is adsorbed.

**Fig. 8:**
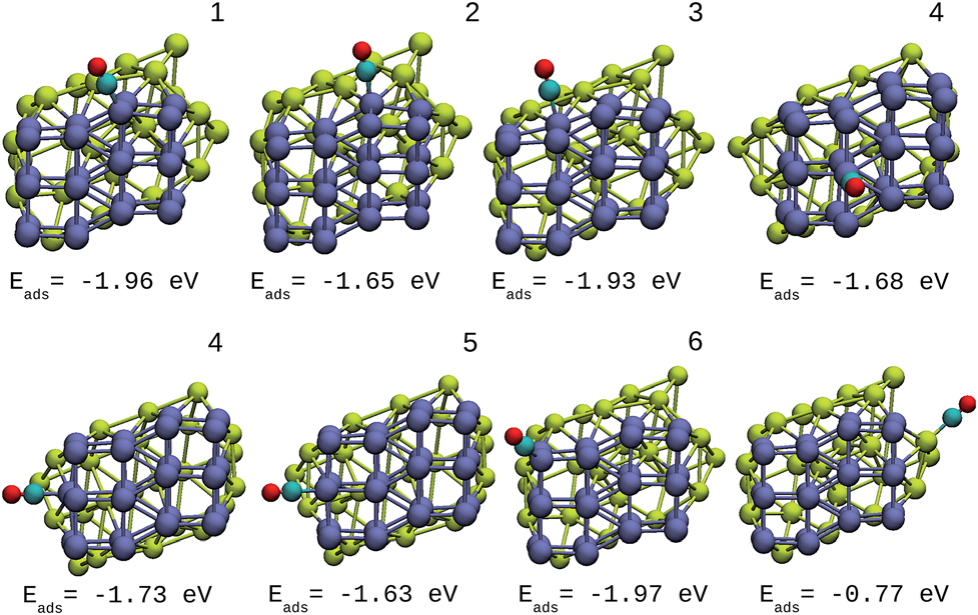
Different positions of molecular CO adsorbed on the most stable Ir_24_Au_24_ NA with CO adsorption energies. The numbers on each structure symbolize the different Ir atomic positions where CO is adsorbed.

**Fig. 9:**
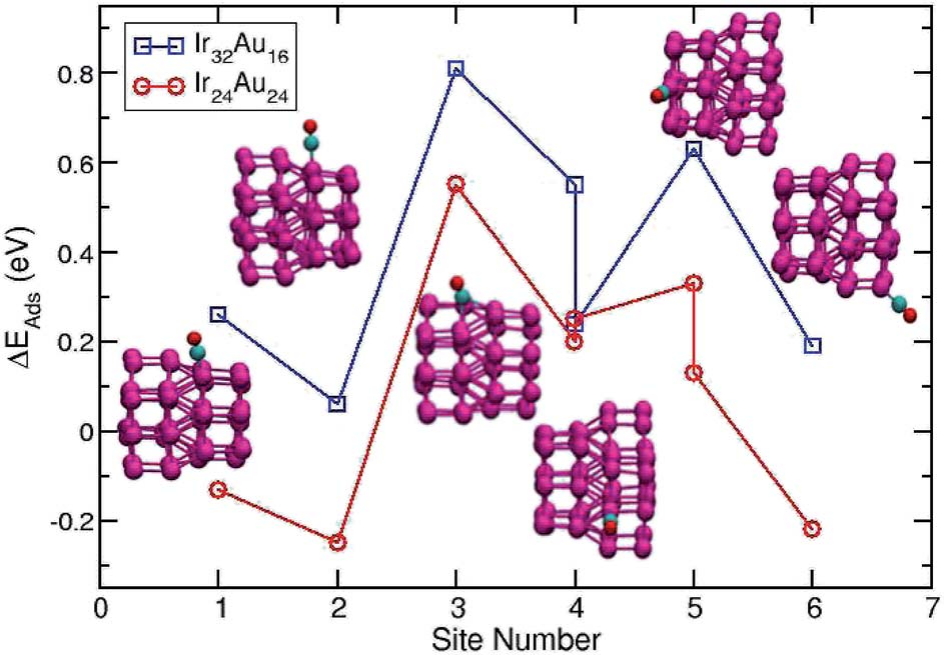
The relative CO adsorption is defined as the difference between the energy of a specific CO adsorption on an Ir atomic site of pure Ir NPs and the energy of CO adsorption on a similar atomic position in each different atomic distribution of IrAu NAs.

A direct comparison between the adsorption energies of CO on the pure and mixture clusters can be misleading since gold often induces changes in the coordination and geometry of Ir ensembles. The changes in the adsorption energy could be attributed to the change in coordination rather than the changes occurring as a consequence of the d-band shift. For this reason, only cases with similar chemical environments were compared. Numbers 1–6 are assigned to each corresponding position in Ir_48_ in Fig. 7 and 8. In this way, sites homologous to Ir_48_ were found but with different chemical environments due to the presence of Au. As in the case shown in Fig. 7, where two 4 sites were recorded, the first has double coordination with Au and has lower energy than the second. On the other hand, the adsorption energies of CO on the Au atoms for the IrAu NAs are always smaller in absolute value, with more than a 1–1.6 eV difference with respect to the adsorption on the Ir atoms. In the IrAu NA with *N* = 48 the values of the CO adsorption energies remain negative and even in some positions more negative than those in Ir_48_. Relative CO adsorption energies (Δ*E*) are defined as the difference between the adsorption energy of CO on pure Ir with respect to that on an analogous site on the IrAu NA. Fig. 9 shows Δ*E* for two IrAu NA compositions. The CO adsorption on Ir atoms close to Au atoms is 0.62 eV more stable than that on Ir atoms far from the Au atom, as in the case of positions 4 and 5 for Ir_32_Au_16_ shown in Fig. 9. At position 6 for Ir_32_Au_16_ the Au atoms are located farther than in position 5, where the Ir atom is adjacent to a Au atom, as shown in Fig. 7. As shown in Fig. 9, in almost all the CO adsorption Ir positions for Ir_32_Au_16_ and Ir_24_Au_24_ the energies are positive, indicating that CO adsorption on these positions is favorable.

Summarizing this section, the presence of Au atoms in the IrAu mixture enhances the adsorption of CO on Ir atoms and this has also been evidenced experimentally.^34^

## 4 Conclusions

In this work, the structural and electronic properties of Ir_*m*_Au_*n*_ NAs with *N* = 8, 27, 48 and 64 were investigated with DFT. This was successful to obtain mixed IrAu NA structures of more than 20 atoms similar to those observed in STEM. Segregation is found in all the mixtures studied where the Ir atoms are in the cluster internal region and the Au atoms are situated on the cluster surface. Ir atoms maintain their structural ordering in the IrAu NAs, in contrast to Au atoms, which are packed in a disordered arrangement. In fact, these results could complement the interpretation of the experimental analysis of STEM images.

PDOS reveals an up-shift of the d-band of Ir induced by Au. According to the d-band model, this fact allows us to find a possible interpretation for the improvement in the reactivity of Ir in the presence of Au, which was confirmed using CO as a probe molecule. These findings open up new possibilities to explore the catalytic potential of mixed IrAu systems, which are of great technological interest.

## Conflicts of interest

There are no conflicts to declare.

## Supplementary Material

RA-008-C7RA13347B-s001
