# Supplementary material for: A density functional study on the reactivity enhancement induced by gold in IrAu nanoalloys
Source: RSC Adv. 2018 Mar 14;8(19):10450–6. doi: 10.1039/c7ra13347b (PMC9078919; doi:10.1039/c7ra13347b)
Supplement: RA-008-C7RA13347B-s001 [file RA-008-C7RA13347B-s001.pdf]

Electronic Supplementary Material (ESI) for RSC Advances.
This journal is © The Royal Society of Chemistry 2018

Supplementary Information

Reactivity Enhancement Induced by Gold in IrAu Nanoalloys

Paula S. Cappellari, Germán J. Soldano and Marcelo M. Mariscal

December 15, 2017

1. IrAu NA structures

IrAu NA 2D nanostructures for N = 9. We started with a 3x3 rectangle of pure Ir and from there are gradually exchanged Ir atoms by Au atoms. As the number of Au atoms in the structure is increased, the square is transformed into a shape similar to 2D structures of pure Au. It should be noted that the simple analysis of this type of structure allowed to evaluate how the IrAu mixed system behaves.

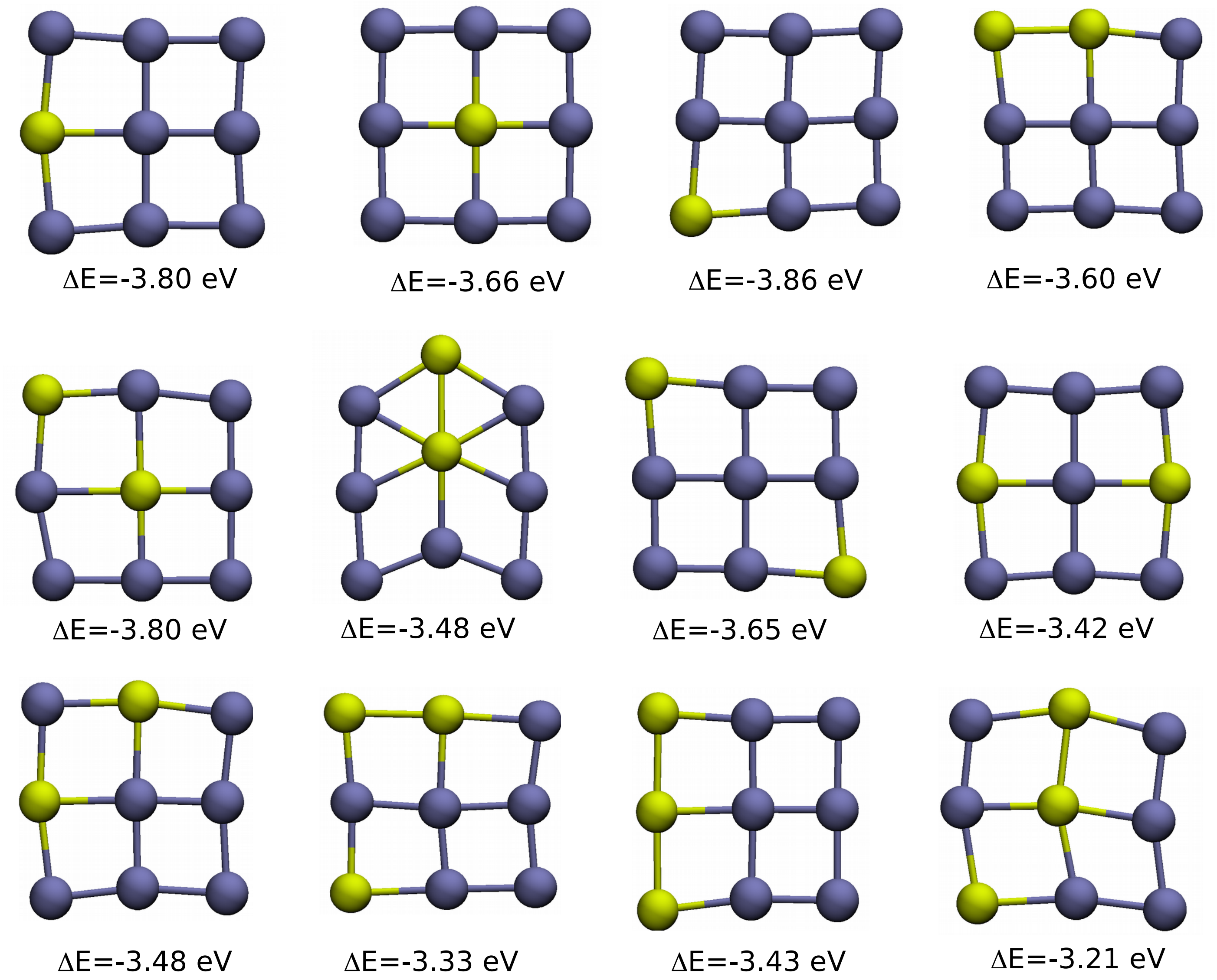

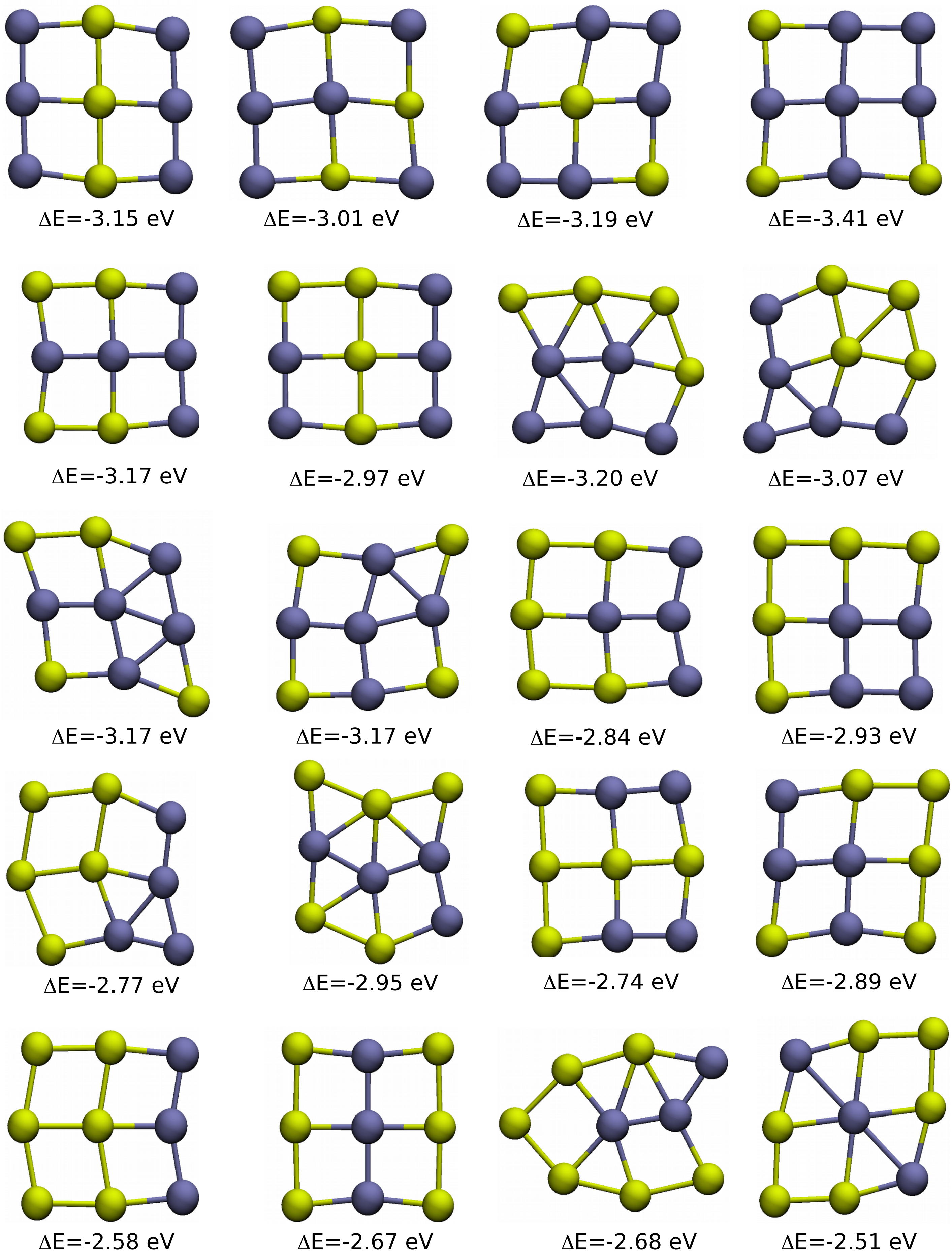

ΔE=-3.15 eV
ΔE=-3.01 eV
ΔE=-3.19 eV
ΔE=-3.41 eV
ΔE=-3.17 eV
ΔE=-2.97 eV
ΔE=-3.20 eV
ΔE=-3.07 eV
ΔE=-3.17 eV
ΔE=-3.17 eV
ΔE=-2.84 eV
ΔE=-2.93 eV
ΔE=-2.77 eV
ΔE=-2.95 eV
ΔE=-2.74 eV
ΔE=-2.89 eV
ΔE=-2.58 eV
ΔE=-2.67 eV
ΔE=-2.68 eV
ΔE=-2.51 eV

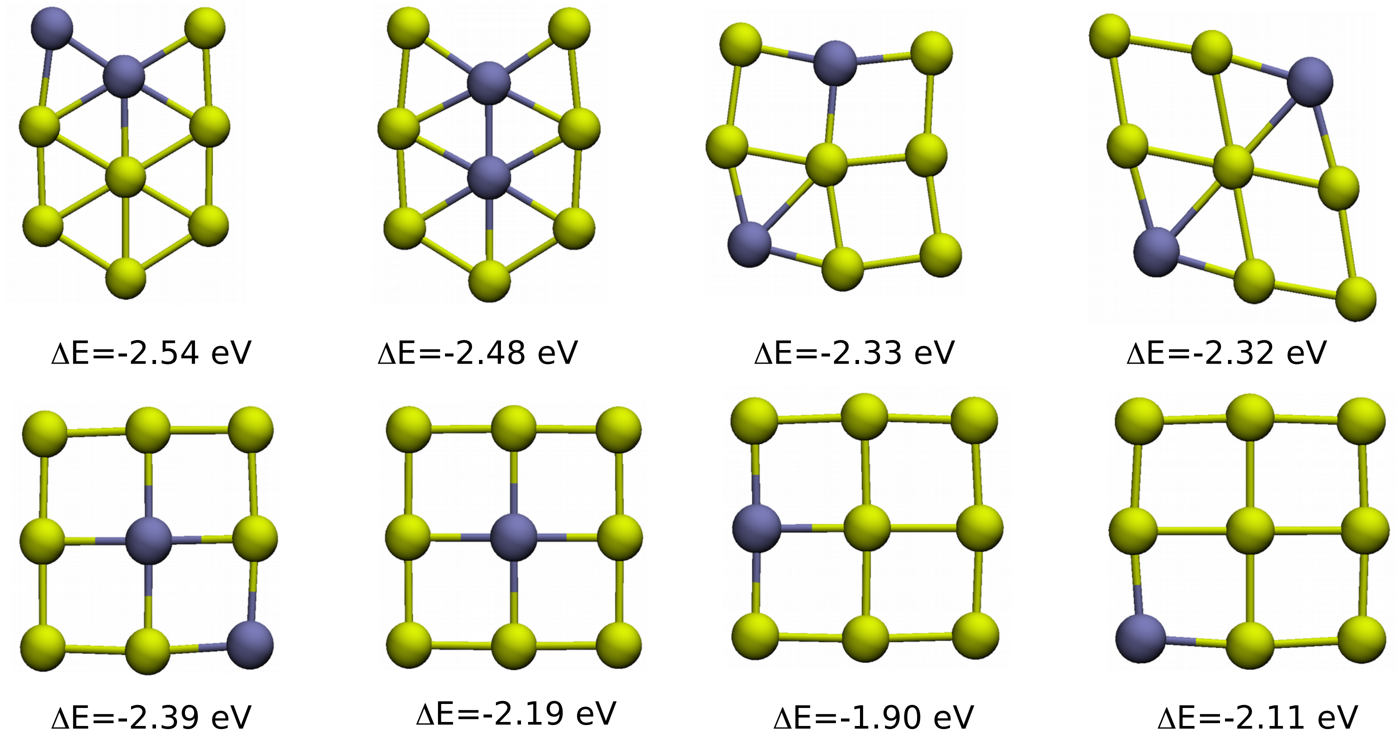

Figure S1. The IrAu NA 2D N = 9. Yellow spheres represent atoms of Au and gray spheres atoms of Ir. Under each structure the formation energy ΔE is reported.

To obtain the different IrAu NA isomers from each atomic distribution with N = 27, we start from the cubic structure pure Ir and it is exchange Ir atoms for Au atoms. In this way, four different structure for each atomic distribution were built. The distribution atomic select here were $Ir_{23}Au_4$, $Ir_{21}Au_6$, $Ir_{19}Au_8$, $Ir_{14}Au_{13}$ and $Ir_{10}Au_{17}$.

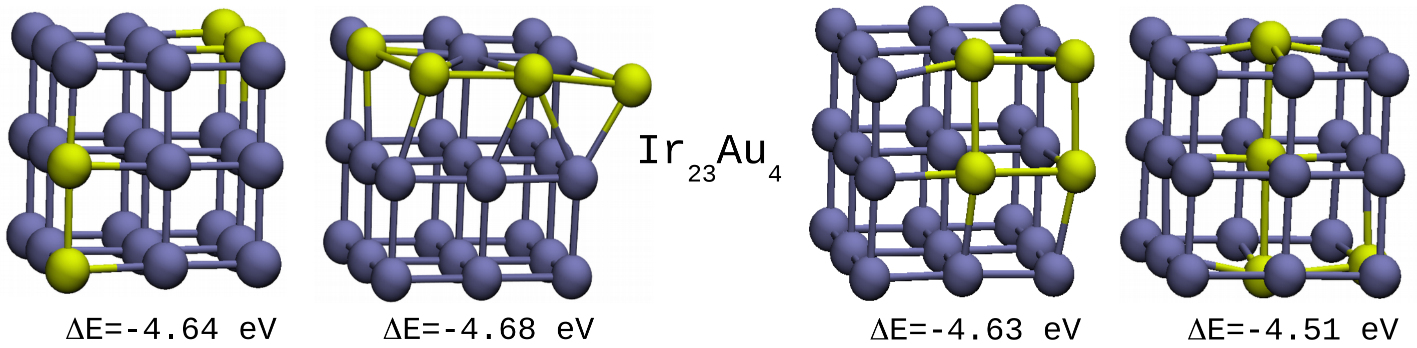

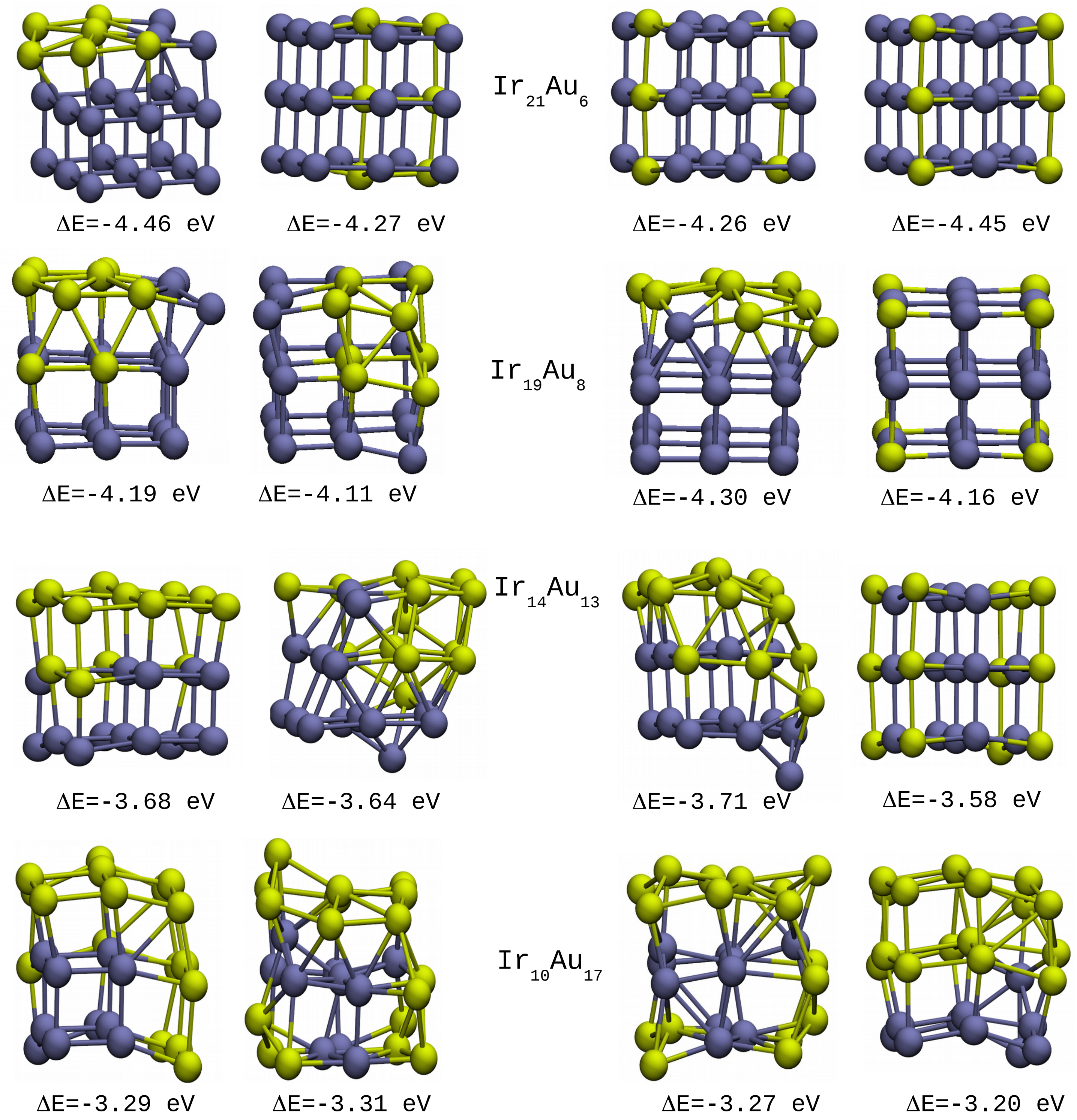

Figure S2. The IrAu NA 3D N = 27. Yellow spheres represent atoms of Au and gray spheres atoms of Ir. Under each structure the formation energy ΔE is reported.

The pair correlation functions *g(r)* was analyzed for the most relevant IrAu NA structures. It has been calculated as a function of distance for the Ir-Ir and Au-Au interactions separately.

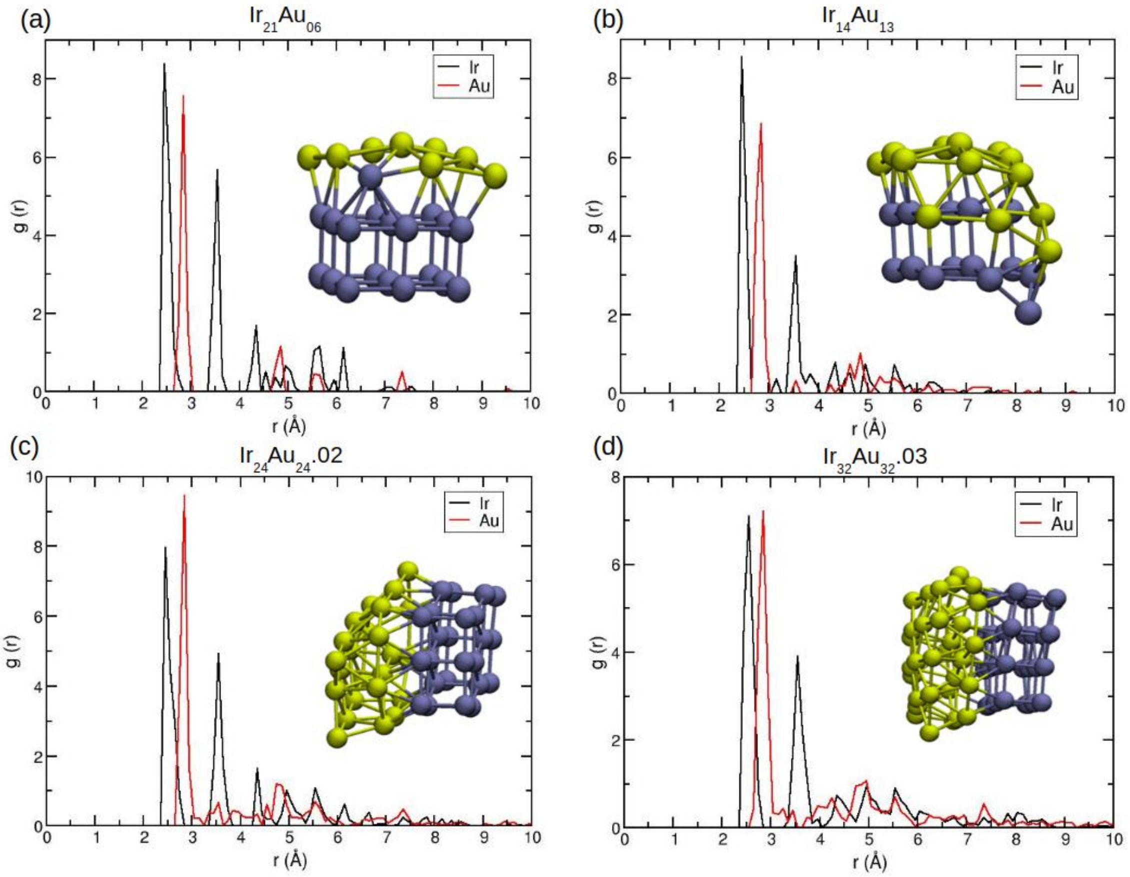

Figure S3. Pair correlation functions for the Ir-Ir and Au-Au interactions in $Ir_{21}Au_{06}$ (a), $Ir_{14}Au_{13}$ (b), $Ir_{24}Au_{24}$ (c) and $Ir_{32}Au_{32}$ (d).

For all the cases studied, Ir presents several well defined peaks up to third neighbors, a clear indication of ordering. Meanwhile, for Au, only the first peak is present, a characteristic of a non-order structure. It is possible to establish that IrAu NA prevails the order of coordination of Ir atoms among them with regard to Au atoms among them. According to the pair correlation function g(r) shown in figure S3, it is observed that the more stable structures present a greater coordination on the part of Ir atoms, as evidenced by the presence of first, second and even third nearest-neighbors given by the sharp peaks.

The first nearest neighbor peak at r = 2.47 Å from Ir atoms, as shown in Figure S3 (a), and r = 2.87 Å from Au atoms in $Ir_{21}Au_{6}$. In this case the contribution of other neighbors to Au is minimal, instead for Ir atoms is detected second neighbor peak at r = 3.56 Å and third neighbors at r = 4.43 Å. This behavior is also evidenced in the other IrAu NAs reported here, Figure S3 (b), (c) and (d).

Furthermore, the Au-Au partial position has its first peak at a distance very close to the Au-Au bond length in pure Au (r= 2.88 Å),[1] r = 2.86 Å for each informed case. It should be mentioned that in the present work the distances of first neighbors for Au bulk 2.93 Å. On the other hand, the distances of first neighbors for Ir bulk 2.75 Å. As for the latter, Ir-Ir bond length that is reported experimentally is 2.71 Å.[2]

In all IrAu NA structures reported here, there is a decrease with respect to pure Ir in the distance of Ir-Ir first neighbors of approximately 0.17 Å. This fact indicates that Ir atoms are closer in IrAu NA structures than in pure Ir, while Au atoms maintain the interatomic mean distance. Based on the above, Ir atoms form a more compact structure than Au atoms in IrAu NA. As can be seen in Fig. S3 (b), the Ir-Ir first peak is positioned at distance of 2.45 and the second peak is located at a distance of 3.56 Å. On the other hand, there is only significant peak at distance of 2.86 Å for Au-Au interaction. Continuing this trend, en Fig. S3 (c), the presence of the first Ir-Ir neighbors is evidenced with an acute peak in 2.46 Å, second neighbors at 3.59 Å and even a peak at 4.37 Å is shown, indicating the presence of third neighbors. Also in Fig. S3 (d) a first peak at 2.55 Å and a second peak at 3.57 Å shows the presence of first and second neighbors to Ir in the $Ir_{32}Au_{32}$ structure, while that at 2.88 Å the presence of first neighbors for Au is recorded. Likewise, The presence of second and third well-defined peaks is observed only for the Ir-Ir interaction, this suggests that the optimization of the IrAu NA structures studied here has prevailed the coordination of the Ir atoms on the Au atoms.

The projected density of states (PDOS) for the most stable structures for IrAu NA with N=27 and N=64 were calculated. In each case they are compared with the PDOS of the pure clusters.

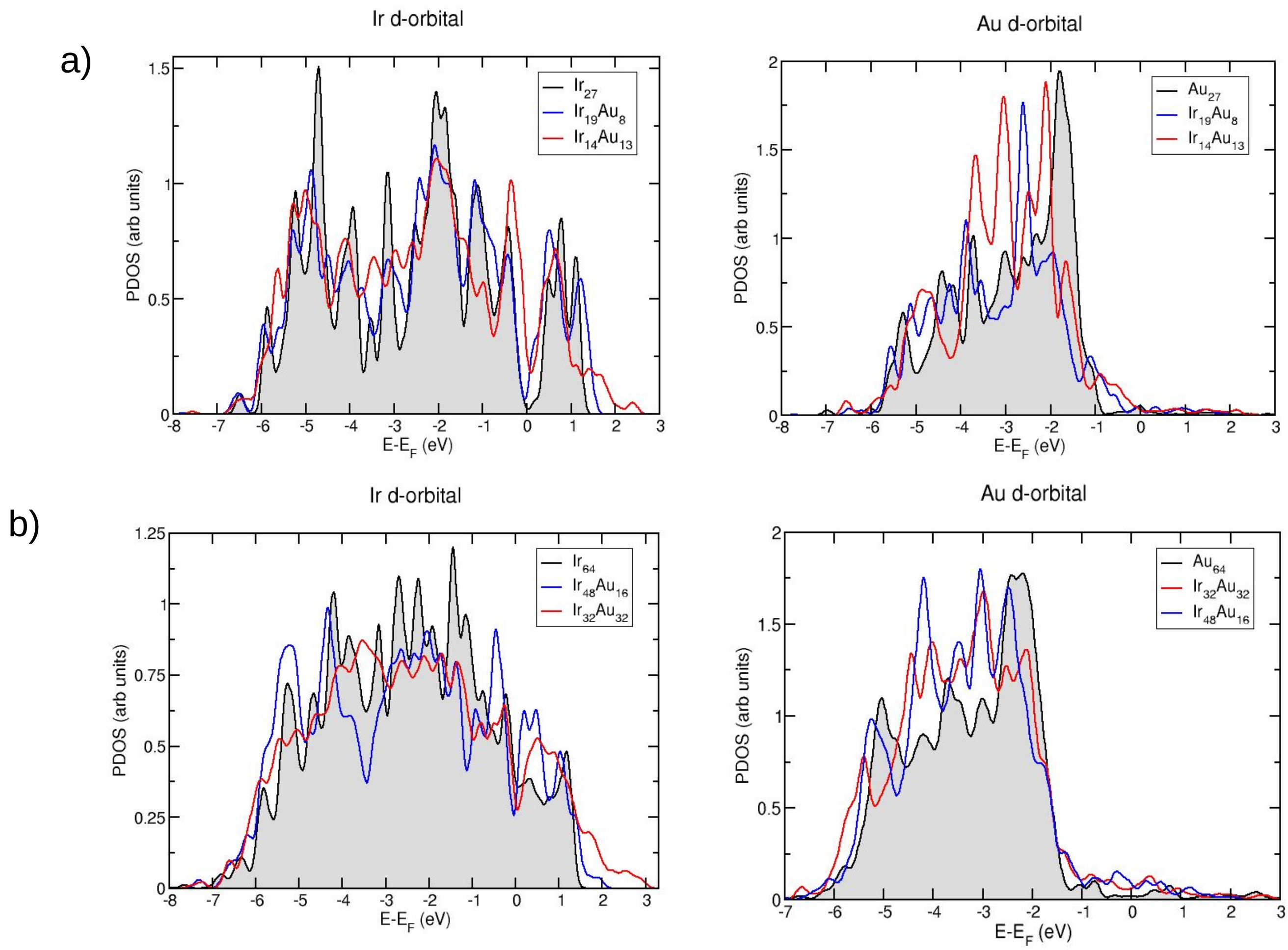

Figure S4. The projected density of electronic states (PDOS) plots d-orbitals (a) of the average Ir atoms and Au atoms on N=27 IrAu NA and (b) the average Ir atoms and Au atoms on N=64 IrAu NA.

The different adsorption atomic site of CO in pure Au_{48} were performed.

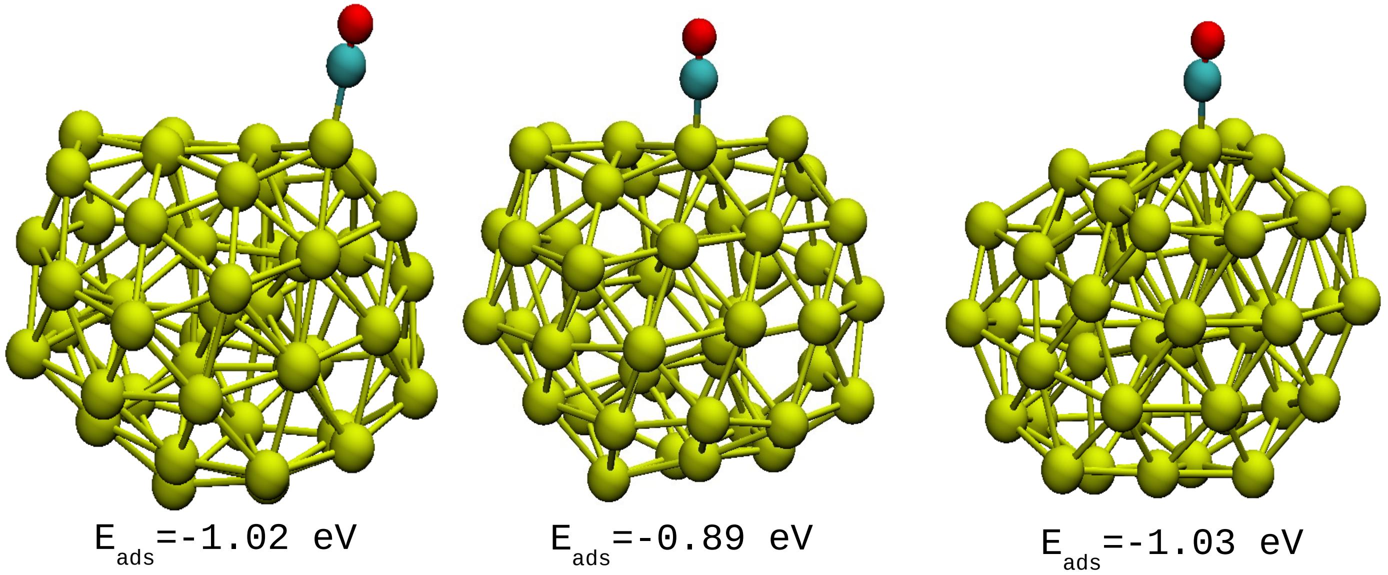

Figure S5. Different positions of molecular CO adsorbed on the most stable Au_{48} cluster with CO adsorption. The numbers on each structure symbolize the energy adsorption.

References

1. J.T.Miller, A.J.Kropf, Y.Zha, J.R.Regalbuto, L.DelannoydC.Louis, E.Bus, J.A. van Bokhoven, *Journal of Catalysis,* 2006, 240, 222-234.

2. I. A. Erikat, B. A. Hamad and J. M. Khalifeh, The European Physical Journal B, 2009, 67, 35-41.

XYZ atomic coordinates of NA (Å)

Ir_{08}

Ir	1.498286	3.445609	8.891485
Ir	2.356581	1.196439	8.374869
Ir	4.601705	2.125400	7.973500
Ir	3.743680	4.375807	8.491454
Ir	0.938510	3.774564	6.514492
Ir	1.785727	1.520928	6.001420
Ir	4.028665	2.452311	5.599307
Ir	3.183232	4.705255	6.116361

Ir_7Au_1

Ir	10.511176	9.340154	12.583018
Ir	12.991272	9.348805	12.442036
Ir	12.982969	11.834731	12.482749
Ir	10.488813	11.833526	12.657667
Ir	10.369696	9.327543	10.091703
Ir	12.864892	9.353634	9.959328
Ir	12.918008	11.846683	9.990376
Au	10.246545	12.004589	9.989138

Ir_6Au_2

Ir	10.506233	9.344891	12.571800
Ir	12.991147	9.326894	12.458596
Ir	12.979744	11.816342	12.512337
Ir	10.507198	11.835498	12.707130
Ir	10.441504	9.460318	10.119488
Ir	12.896942	9.317192	9.977942
Au	12.883369	11.980253	9.868342
Au	10.039120	12.106078	10.107161

Ir_5Au_3

Ir	5.508705	4.136638	5.478110
Ir	7.462939	4.448301	7.259122
Ir	4.980093	6.819831	7.670098
Ir	5.350521	6.584647	5.257539
Ir	5.040088	4.353467	7.973806
Au	8.059930	4.298703	4.619562
Au	7.823844	7.131247	4.517702
Au	7.632413	7.112634	7.384023

Ir_4Au_4

Ir	10.754730	9.238649	12.579661
Ir	13.200326	9.324452	12.932776
Ir	13.151225	11.713127	12.300472
Ir	10.683489	11.675300	12.113096
Au	10.225363	9.695337	9.962187
Au	12.958110	9.264418	10.277854
Au	12.378384	11.930834	9.708851
Au	9.581575	12.470819	9.825737

Ir_3Au_5

Ir	8.133682	4.541122	4.913657
Ir	7.561424	4.216733	7.307795
Ir	7.215497	6.610137	6.877485
Au	5.228563	7.364745	5.035277
Au	4.916215	4.210844	8.034266
Au	5.500746	4.604710	5.289043
Au	8.017771	7.195201	4.403374
Au	4.691584	6.974694	7.762428

Ir_2Au_6

Ir	10.264433	9.313022	10.314726
Ir	12.576722	9.896398	9.839402
Au	12.963293	12.127508	11.714045
Au	10.686405	12.006285	9.928038
Au	11.686864	9.296481	12.622368
Au	14.313830	9.720430	11.864792
Au	13.299425	12.305195	8.907243
Au	10.338835	11.700774	12.614700

Ir_1Au_7

Ir	10.915694	9.322489	12.815028
Au	13.484136	8.643777	13.244766
Au	12.947447	11.298547	12.497772
Au	10.355634	11.877415	13.395882
Au	9.973444	8.455272	10.448299
Au	12.695221	9.068926	10.589165
Au	13.168174	11.670851	9.772969
Au	10.607356	11.178278	10.690444

Au_{08}

Au	2.819467	3.200275	7.710267
Au	4.465466	3.451342	5.359537
Au	2.889886	0.323090	7.609698
Au	4.496246	0.657140	5.351828
Au	5.482247	2.124540	7.510099
Au	1.882106	4.431580	5.509077
Au	3.880083	1.785520	9.738547
Au	1.896085	1.665987	5.456669

Ir_{27}

Ir	10.818807	11.100792	8.864580
Ir	8.414317	9.024896	10.421871
Ir	11.334260	12.193385	11.056786
Ir	13.311190	8.656803	9.463060
Ir	13.782094	11.974538	10.592011
Ir	9.404506	13.391783	13.761047
Ir	11.948514	8.697917	15.434389
Ir	13.262408	10.913206	8.389348
Ir	13.868446	9.713767	11.660925
Ir	10.856557	8.824474	9.908093
Ir	8.884935	12.341325	11.556327
Ir	11.851064	13.215012	13.280574
Ir	11.382428	9.899191	12.149709
Ir	10.911098	6.584408	11.018835
Ir	11.908327	10.973726	14.391582
Ir	13.357539	6.403245	10.542543
Ir	8.464998	6.773447	11.497197
Ir	9.503641	8.880259	15.909902
Ir	8.895407	10.084308	12.638990
Ir	13.879240	7.458175	12.745177
Ir	8.367847	11.285206	9.345034
Ir	14.399879	8.517589	14.954108
Ir	14.349770	10.777250	13.875766
Ir	14.296226	13.029785	12.798535
Ir	11.430433	7.606078	13.242319
Ir	9.452388	11.137251	14.838358
Ir	8.981163	7.824350	13.705767

$Ir_{23}Au_4$

Ir	10.731581	11.076604	8.897708
Ir	9.048175	10.044774	12.741456
Ir	11.328779	12.160277	11.070398
Ir	13.353741	8.730102	9.332093
Ir	13.751560	12.041501	10.463128
Ir	9.547352	13.282275	13.876237
Ir	8.489610	8.962803	10.580760
Ir	13.146137	10.971907	8.289841
Ir	13.969315	9.838483	11.468539
Ir	10.899252	8.830699	9.949003
Ir	8.904982	12.275060	11.692089
Ir	11.957875	13.189184	13.276545
Ir	11.494642	9.914884	12.124665
Ir	11.088649	6.589384	11.125946
Ir	12.126882	11.020611	14.350066
Ir	13.327851	6.364346	10.097636
Ir	8.705397	6.754493	11.700958
Ir	8.321463	11.204211	9.509603
Au	11.187750	8.773269	15.803421
Au	14.256920	7.264421	12.455528
Au	8.338641	9.033726	16.029403
Au	13.803891	8.529576	14.841447
Ir	14.507473	10.846350	13.667774
Ir	14.353644	13.092362	12.630836
Ir	11.649911	7.604559	13.262497

Ir	9.675184	11.086820	14.955870
Ir	9.261018	7.786036	13.844615

$Ir_{21}Au_6$

Ir	10.635407	11.080762	8.761052
Ir	8.371325	9.002424	10.393649
Ir	11.261161	12.134483	10.933532
Ir	13.345065	9.064195	10.276579
Ir	8.238077	11.260458	9.382377
Ir	9.559859	13.371994	13.691640
Ir	12.062349	8.666233	15.257940
Ir	13.038305	10.572855	8.350392
Ir	8.980007	10.079302	12.605794
Ir	10.715796	8.754858	9.764432
Ir	8.911990	12.315586	11.536221
Ir	11.951924	13.148378	13.105286
Ir	11.373494	9.855487	12.023797
Ir	10.735586	6.514879	10.954157
Ir	11.967577	10.933358	14.237571
Ir	13.107912	6.607458	10.208354
Ir	8.338801	6.801729	11.531226
Ir	9.672119	8.894631	15.845200
Au	14.445498	10.161553	12.580120
Au	14.050399	7.346925	12.572036
Au	13.949006	11.953472	10.421990
Au	14.681481	8.504994	14.982263
Au	14.518258	11.336602	15.123521
Au	14.580855	13.056945	12.863057
Ir	11.370536	7.576967	13.119439
Ir	9.627602	11.142228	14.786016
Ir	9.020163	7.847658	13.686263

$Ir_{19}Au_8$

Ir	9.347432	13.336082	13.824348
Ir	11.848120	10.916753	14.396890
Ir	11.163690	12.078298	11.140261
Au	10.291599	11.509975	8.600280
Ir	13.555684	11.804365	10.533470
Ir	11.757331	13.131156	13.238385
Ir	11.953935	8.667754	15.542684
Au	12.834912	10.591080	8.247103
Ir	13.716390	9.623065	11.705431
Ir	9.508971	7.842112	11.528958
Ir	8.729804	12.275306	11.703872
Au	7.752134	12.490909	9.267194
Ir	11.267867	9.828204	12.278148
Ir	14.365230	8.508639	14.934367
Au	10.887327	8.792663	9.294375
Au	11.822422	6.595500	10.810631
Ir	9.553089	8.938101	16.170708
Ir	8.895152	7.775411	14.079763
Ir	8.766283	10.055318	12.863060
Au	14.568395	5.975015	10.732331
Ir	14.164525	12.926758	12.672203
Ir	13.813619	7.385964	12.825924

Ir	14.267876	10.717737	13.794358
Au	13.710218	8.245090	9.331667
Ir	11.389516	7.532532	13.437082
Ir	9.398303	11.128075	14.979988
Au	8.389299	9.863479	10.076658

$Ir_{14}Au_{13}$

Ir	11.028938	9.149185	9.993081
Ir	13.439274	9.009851	9.371987
Ir	9.119899	10.396445	12.715514
Ir	11.594941	10.343411	12.172631
Ir	13.600369	6.796550	10.561167
Ir	8.763261	7.123814	11.664125
Ir	11.180907	7.004096	11.104447
Ir	13.957569	6.905515	15.206091
Ir	9.885050	8.885517	16.108988
Ir	9.307865	8.239933	13.793096
Ir	11.756405	8.098046	13.261879
Ir	14.212316	7.524556	12.803861
Ir	8.603099	9.272129	10.525316
Ir	12.274761	8.665672	15.687903
Au	13.239560	11.800298	10.092957
Au	8.144132	10.627645	8.299767
Au	10.707443	11.708699	8.827461
Au	12.529372	11.275031	15.231373
Au	13.012389	10.874222	7.495954
Au	8.532659	12.167906	10.574922
Au	11.061878	13.074267	11.373407
Au	14.685258	9.580919	14.615495
Au	11.303532	13.507888	14.151115
Au	9.690308	11.399848	15.245632
Au	13.478045	12.363304	12.810119
Au	8.727559	13.095127	13.162594
Au	14.527749	9.982493	11.767852

Au_{27}

Au	10.826066	10.698436	12.867481
Au	7.744244	13.019954	8.653327
Au	7.709541	8.456576	7.378436
Au	8.172160	11.109438	6.546319
Au	8.655555	12.472877	13.008403
Au	10.045649	7.106010	8.455259
Au	12.749528	10.893602	8.205660
Au	11.155457	13.360104	11.869544
Au	7.515963	6.427117	9.288793
Au	9.721925	5.602237	10.838807
Au	6.293688	10.653633	8.546480
Au	8.241028	9.115812	10.065823
Au	9.588949	11.599498	10.322126
Au	12.956446	11.329788	11.091541
Au	12.584132	8.005289	7.785870
Au	6.866038	11.611394	11.057854
Au	10.403860	12.422163	7.638163
Au	12.192739	6.761440	10.524657

Au	9.887945	14.473186	9.556225
Au	8.084221	9.742577	12.848917
Au	12.439157	13.425466	9.306504
Au	11.106298	9.344373	10.031367
Au	10.209946	7.911453	12.446723
Au	14.046720	8.910078	9.966179
Au	7.607096	7.119105	12.026885
Au	10.377309	9.626349	7.213545
Au	12.818340	8.802057	12.459107

Ir_{48}

Ir	7.918149	5.534240	4.301406
Ir	10.414226	5.525080	4.328896
Ir	12.630851	5.304976	5.728266
Ir	15.086009	5.314886	5.943766
Ir	7.916105	8.030257	4.348081
Ir	10.400270	8.009592	4.296209
Ir	12.643875	7.767612	5.690548
Ir	15.105852	7.772196	5.754270
Ir	7.715758	10.236662	5.766948
Ir	10.177931	10.242658	5.710059
Ir	12.425426	10.026075	4.317585
Ir	14.909479	10.004185	4.375767
Ir	7.734509	12.689960	6.003086
Ir	10.190278	12.704184	5.794082
Ir	12.410850	12.509541	4.396562
Ir	14.906886	12.500575	4.374590
Ir	7.792348	5.389067	6.792092
Ir	10.280057	5.287680	6.896959
Ir	12.464031	5.347726	8.349568
Ir	14.937021	5.439013	8.446421
Ir	7.699117	7.876246	6.916677
Ir	10.245325	7.833726	6.943668
Ir	12.476165	7.907926	8.381726
Ir	15.036706	7.912554	8.376475
Ir	7.777656	10.048071	8.386377
Ir	10.338078	10.052905	8.398678
Ir	12.572927	10.153374	6.968339
Ir	15.119267	10.111134	6.947450
Ir	7.876504	12.519770	8.503369
Ir	10.349778	12.613264	8.414580
Ir	12.537853	12.699821	6.968906
Ir	15.025796	12.600176	6.867887
Ir	7.651888	5.228717	9.290187
Ir	10.113460	5.215755	9.515748
Ir	12.343178	5.497337	10.917732
Ir	14.834031	5.516977	10.940773
Ir	7.647095	7.688394	9.534334
Ir	10.098802	7.666325	9.649918
Ir	12.376642	7.980303	11.030720
Ir	14.860807	8.007861	10.943977
Ir	7.946828	9.905115	10.952272
Ir	10.430727	9.932054	11.046176
Ir	12.711914	10.271151	9.677523
Ir	15.163784	10.250941	9.568231
Ir	7.972396	12.395504	10.996187
Ir	10.463456	12.416698	10.979863

Ir	12.697202	12.723739	9.589083
Ir	15.159315	12.714750	9.368848

$Ir_{32}Au_{16}$.01

Ir	7.832666	10.246417	5.106203
Ir	10.316428	10.283559	5.136146
Ir	12.932264	10.282300	5.132163
Ir	15.417960	10.246746	5.106077
Ir	7.836480	12.763522	5.105563
Ir	10.353566	12.767294	5.106195
Ir	12.896713	12.768021	5.106087
Ir	15.413454	12.763475	5.106073
Ir	7.836504	5.186545	5.106072
Ir	10.353248	5.181987	5.106083
Ir	12.896396	5.182720	5.106199
Ir	15.413480	5.186489	5.105563
Ir	7.832014	7.703270	5.106078
Ir	10.317712	7.667707	5.132157
Ir	12.933549	7.666454	5.136155
Ir	15.417307	7.703595	5.106204
Ir	7.821667	5.171676	7.555337
Ir	10.350648	5.147810	7.553812
Ir	12.899264	5.148006	7.554235
Ir	15.428211	5.171783	7.555511
Ir	7.797829	7.700684	7.553828
Ir	10.308760	7.658773	7.562349
Ir	12.941815	7.658172	7.561259
Ir	15.452012	7.700710	7.554219
Ir	7.797977	10.249300	7.554219
Ir	10.308170	10.291839	7.561250
Ir	12.941225	10.291231	7.562354
Ir	15.452161	10.249327	7.553826
Ir	7.821765	12.778224	7.555512
Ir	10.350707	12.802003	7.554229
Ir	12.899322	12.802192	7.553818
Ir	15.428307	12.778335	7.555338
Au	7.558489	4.908426	10.172206
Au	10.299938	4.826203	10.263452
Au	12.950093	4.825904	10.263360
Au	15.691531	4.908482	10.172491
Au	7.476251	7.649899	10.263458
Au	10.270435	7.620419	10.423459
Au	12.979456	7.620560	10.423152
Au	15.774121	7.649918	10.263357
Au	7.475953	10.300066	10.263367
Au	10.270577	10.329430	10.423156
Au	12.979598	10.329569	10.423458
Au	15.773823	10.300082	10.263447
Au	7.558523	13.041502	10.172501
Au	10.299957	13.124070	10.263363
Au	12.950113	13.123768	10.263458
Au	15.691569	13.041550	10.172201

$Ir_{32}Au_{16}$.02

Ir	8.016341	5.634551	4.084435
Ir	10.486225	5.590699	4.292802
Ir	12.576246	5.365916	5.849588
Ir	15.000881	5.398702	6.088914
Ir	7.982351	8.102785	4.312624
Ir	10.445383	8.053648	4.333721
Ir	12.561849	7.848826	5.880458
Ir	15.044953	7.825777	5.876290
Ir	7.776168	10.181155	5.887278
Ir	10.259197	10.158572	5.898575
Ir	12.379687	9.982022	4.353996
Ir	14.842785	9.933095	4.339641
Ir	7.818765	12.603748	6.144874
Ir	10.244238	12.641675	5.913673
Ir	12.338970	12.445324	4.359316
Ir	14.809381	12.405116	4.157229
Ir	7.717976	5.318139	6.505102
Ir	10.178417	5.250656	6.827327
Ir	12.265187	5.423269	8.442730
Ir	14.745064	5.628971	8.547614
Ir	7.661245	7.776181	6.847274
Ir	10.176864	7.764149	7.113186
Ir	12.363715	8.020294	8.574995
Ir	14.962256	8.109091	8.471717
Ir	7.852618	9.849782	8.477381
Ir	10.450707	9.938586	8.589975
Ir	12.641039	10.220755	7.138868
Ir	15.157156	10.212936	6.880595
Ir	8.067226	12.328201	8.599649
Ir	10.547285	12.537739	8.505966
Ir	12.639526	12.738865	6.900332
Ir	15.101010	12.677031	6.584224
Au	6.883944	4.467344	8.844940
Au	9.560036	4.874577	9.441000
Au	11.742377	5.281904	11.082796
Au	14.423654	5.924642	11.189835
Au	7.304716	7.137465	9.458690
Au	9.998052	7.561496	10.134550
Au	12.404347	7.952239	11.597205
Au	15.082446	8.603142	11.118383
Au	7.730176	9.305585	11.114057
Au	10.406143	9.955722	11.612494
Au	12.814865	10.371089	10.160104
Au	15.512235	10.800264	9.503863
Au	8.380684	11.984288	11.236921
Au	11.060393	12.635780	11.149779
Au	13.246606	13.072392	9.522025
Au	15.925228	13.482570	8.943876

$Ir_{24}Au_{24}$.01

Ir	7.814330	10.343557	5.186028
Ir	10.321216	10.380686	5.165496
Ir	12.928806	10.380695	5.165502
Ir	15.435695	10.343550	5.186035
Ir	7.820717	12.823169	5.171770
Ir	10.334549	12.820772	5.158701
Ir	12.915489	12.820775	5.158705

Ir	15.429325	12.823164	5.171771
Au	7.584330	5.028367	4.951171
Au	10.291515	5.011056	4.901503
Au	12.958514	5.011053	4.901500
Au	15.665664	5.028349	4.951168
Au	7.508240	7.748946	4.864736
Au	10.275719	7.754548	4.697857
Au	12.974229	7.754550	4.697859
Au	15.741672	7.748929	4.864726
Au	7.512280	4.976676	7.650048
Au	10.277986	4.760363	7.649890
Au	12.972013	4.760357	7.649889
Au	15.737680	4.976664	7.650050
Au	7.386055	7.738304	7.650023
Au	10.239827	7.634619	7.649978
Au	13.010118	7.634618	7.649979
Au	15.863914	7.738292	7.650020
Ir	7.794271	10.393335	7.650011
Ir	10.307565	10.399168	7.650005
Ir	12.942453	10.399170	7.650008
Ir	15.455741	10.393325	7.650011
Ir	7.811783	12.838861	7.650012
Ir	10.305264	12.836530	7.650008
Ir	12.944763	12.836555	7.650010
Ir	15.438241	12.838853	7.650011
Au	7.584320	5.028365	10.348944
Au	10.291514	5.011063	10.398277
Au	12.958514	5.011063	10.398278
Au	15.665673	5.028352	10.348951
Au	7.508244	7.748946	10.435349
Au	10.275726	7.754538	10.602119
Au	12.974229	7.754543	10.602120
Au	15.741674	7.748933	10.435353
Ir	7.814334	10.343549	10.113994
Ir	10.321220	10.380680	10.134509
Ir	12.928805	10.380693	10.134516
Ir	15.435694	10.343547	10.113988
Ir	7.820720	12.823163	10.128251
Ir	10.334552	12.820768	10.141310
Ir	12.915489	12.820773	10.141314
Ir	15.429324	12.823162	10.128247

$Ir_{24}Au_{24}$.02

Ir	8.642205	5.527194	7.287223
Ir	11.130970	5.707908	7.418146
Ir	13.587679	4.723728	7.636587
Ir	16.055971	5.109403	7.798213
Ir	8.302858	8.079687	7.395389
Ir	10.821578	8.419711	7.469041
Ir	13.220223	7.337255	7.595615
Ir	15.705593	7.622851	7.781067
Ir	7.988022	10.554229	7.558825
Ir	10.482300	10.998305	7.610435
Ir	12.937352	9.986728	7.688441
Ir	15.427046	10.127554	7.875205
Au	7.688416	13.195741	8.457946
Au	9.911166	13.804803	6.756814

Au	12.457543	13.189293	7.749335
Au	14.947858	12.676422	8.804245
Ir	8.544882	5.493236	9.709872
Ir	10.994107	5.714872	9.869396
Ir	13.400937	4.748811	10.048833
Ir	15.855966	5.150203	10.264631
Ir	8.265455	7.972991	9.829054
Ir	10.716732	8.339715	9.968516
Ir	13.078153	7.298386	10.088565
Ir	15.541406	7.628191	10.225702
Ir	7.933522	10.418526	10.014464
Ir	10.405470	10.929625	10.099111
Ir	12.793090	9.883723	10.168648
Ir	15.245279	10.097256	10.330863
Au	8.345042	12.815927	11.216533
Au	10.336992	13.698306	9.495291
Au	12.587620	12.599176	10.683395
Au	15.154992	12.434591	11.630602
Au	7.887867	4.142761	5.188338
Au	10.234493	5.720067	4.692837
Au	12.651719	4.524529	5.081085
Au	15.150265	3.427181	5.714466
Au	7.712999	6.949276	4.908228
Au	10.099767	8.530688	4.573248
Au	12.612676	7.351990	4.681131
Au	15.105426	6.263726	5.336709
Au	7.527484	9.706168	4.986885
Au	9.873250	11.273245	4.576863
Au	12.397679	10.188419	4.775212
Au	14.850214	9.049659	5.387751
Au	7.602819	12.491907	5.703793
Au	9.430389	13.981633	4.072679
Au	12.047461	13.052575	4.939497
Au	14.309071	11.861822	6.055277

Au_{48}

Au	8.340962	14.830498	12.217219
Au	12.191518	8.267377	9.781345
Au	11.103347	10.123460	13.696201
Au	7.339654	6.523027	9.896420
Au	7.120033	12.755834	13.824549
Au	12.222532	7.568161	13.381642
Au	6.037599	9.045547	9.723713
Au	9.992895	15.578336	10.035898
Au	7.453457	14.499725	9.438725
Au	13.444956	9.745733	12.076853
Au	8.303708	10.227902	13.709647
Au	10.072603	7.454969	6.060625
Au	8.360942	10.509669	9.014480
Au	9.466770	7.716880	14.112292
Au	8.364983	12.066663	11.322248
Au	11.460466	9.915255	5.463029
Au	8.719403	5.468654	7.583107
Au	9.900194	12.687149	13.714764
Au	11.499652	5.604927	7.901572
Au	10.014467	7.820848	11.447608
Au	10.982952	5.204607	12.716970

Au	14.153088	7.021362	11.504464
Au	13.615245	11.586699	9.934037
Au	12.427781	14.143256	10.145916
Au	6.103172	10.374055	7.158093
Au	10.058567	12.819010	9.267642
Au	5.867662	12.052681	9.344677
Au	9.462037	14.776377	7.386049
Au	7.555999	12.753387	7.181048
Au	14.121565	6.456371	8.682594
Au	5.931835	10.790834	11.971018
Au	8.706918	9.979540	6.13249
Au	12.487112	12.828203	7.692439
Au	11.021620	10.347160	8.158628
Au	11.120069	14.849236	12.507997
Au	8.288512	5.727239	12.382908
Au	14.843980	9.058117	9.600728
Au	9.654188	7.903862	8.779941
Au	12.409096	12.352234	12.422071
Au	12.521083	5.016599	10.454200
Au	9.761701	4.916942	10.108073
Au	7.234360	7.845898	7.328266
Au	5.827649	13.661207	11.547511
Au	10.183521	12.386084	6.108376
Au	10.688436	10.523985	10.919057
Au	12.804412	7.716849	6.608453
Au	13.697276	10.273143	7.366186
Au	7.060021	8.224451	12.188233

Ir_{64}

Ir	7.920608	5.548007	4.355819
Ir	10.408425	5.526792	4.291886
Ir	12.639273	5.279889	5.648279
Ir	15.111133	5.283105	5.861924
Ir	7.912646	8.036275	4.310866
Ir	10.412635	8.027628	4.208229
Ir	12.674212	7.736226	5.557841
Ir	15.130311	7.756643	5.668822
Ir	7.689016	10.257089	5.686293
Ir	10.144679	10.279586	5.577773
Ir	12.405430	10.009078	4.224337
Ir	14.905280	9.998280	4.327549
Ir	7.707458	12.727344	5.918042
Ir	10.179541	12.734435	5.706079
Ir	12.409791	12.508013	4.345992
Ir	14.897671	12.485520	4.410866
Ir	7.842262	5.449417	6.887641
Ir	10.320053	5.376372	6.905843
Ir	12.544415	5.296273	8.304088
Ir	15.021585	5.353748	8.396897
Ir	7.782635	7.927546	6.925219
Ir	10.316948	7.910811	6.891216
Ir	12.578634	7.811056	8.304816
Ir	15.093621	7.831402	8.324916
Ir	7.725733	10.140929	8.341508
Ir	10.240486	10.161590	8.322614
Ir	12.502099	10.084081	6.908555
Ir	15.036193	10.067009	6.943789

Ir	7.797638	12.617215	8.451699
Ir	10.274934	12.676326	8.360975
Ir	12.499473	12.618122	6.962421
Ir	14.977315	12.545368	6.943519
Ir	7.775089	5.361658	9.452363
Ir	10.251903	5.290374	9.540877
Ir	12.477070	5.338109	10.940272
Ir	14.955432	5.398935	10.963637
Ir	7.717741	7.838208	9.559872
Ir	10.231509	7.804248	9.578228
Ir	12.394195	7.950486	13.674904
Ir	15.029812	7.877036	10.961574
Ir	7.787669	10.052571	10.976030
Ir	10.323051	10.056033	11.008048
Ir	12.586962	10.147543	9.597248
Ir	15.100580	10.113431	9.580493
Ir	7.861652	12.530486	11.017297
Ir	10.340260	12.591725	10.996731
Ir	12.566832	12.661292	9.598996
Ir	15.044012	12.591292	9.509640
Ir	7.682335	5.250950	11.985587
Ir	10.154560	5.230774	12.194460
Ir	12.382873	5.451909	13.556301
Ir	14.870337	5.463164	13.496866
Ir	7.676999	7.721344	12.213102
Ir	10.133329	7.686320	12.319002
Ir	12.494188	7.873419	10.991916
Ir	14.892640	7.950474	13.577194
Ir	7.921157	9.937468	13.590605
Ir	10.421025	9.935217	13.689552
Ir	12.682775	10.221814	12.340014
Ir	15.138998	10.188267	12.235135
Ir	7.944307	12.424731	13.549214
Ir	10.432085	12.436464	13.610028
Ir	12.662098	12.678760	12.252940
Ir	15.133615	12.661706	12.045021

$Ir_{48}Au_{16}$

Ir	7.989516	5.605978	4.236201
Ir	10.479087	5.565770	4.269478
Ir	12.691915	5.244416	5.686032
Ir	15.155273	5.241402	5.905987
Ir	7.979552	8.096234	4.294417
Ir	10.464234	8.055019	4.264360
Ir	12.712635	7.707194	5.632733
Ir	15.174948	7.707906	5.699705
Ir	7.673158	10.291145	5.750617
Ir	10.136054	10.298951	5.659095
Ir	12.389800	9.970698	4.280968
Ir	14.875951	9.936907	4.294419
Ir	7.688083	1.,752602	6.004754
Ir	10.151214	12.762047	5.770491
Ir	12.358743	12.454023	4.328865
Ir	14.850765	12.429705	4.286462
Ir	7.869008	5.455153	6.754083
Ir	10.347757	5.373707	6.845552
Ir	12.518794	5.289470	8.331198

Ir	15.001523	5.376125	8.406758
Ir	7.800159	7.931930	6.876309
Ir	10.337489	7.909353	6.914669
Ir	12.566235	7.830361	8.370617
Ir	15.108515	7.859337	8.344082
Ir	7.43256	10.091022	8.392398
Ir	10.283572	10.126077	8.391168
Ir	12.513194	10.072548	6.931179
Ir	15.047789	10.045425	6.876285
Ir	7.846219	12.571870	8.500474
Ir	10.329524	12.666036	8.410507
Ir	12.502714	12.610114	6.911629
Ir	14.980812	12.525595	6.805160
Ir	7.705912	5.265653	9.267588
Ir	10.168862	5.242377	9.433189
Ir	12.335206	5.412960	10.961491
Ir	14.844329	5.517372	10.893168
Ir	7.682893	7.726362	9.464409
Ir	10.164942	7.717788	9.603306
Au	12.350519	7.987921	14.021764
Ir	14.965014	8.021340	10.972550
Ir	7.873449	9.874166	11.017329
Ir	10.437971	9.950076	11.033612
Ir	12.682841	10.222121	9.618410
Ir	15.161079	10.198922	9.467765
Ir	8.014418	12.382696	10.984621
Ir	10.520832	12.515128	11.037587
Ir	12.685548	12.700435	9.502696
Ir	15.148793	12.664239	9.318866
Au	6.732300	4.483720	11.642528
Au	9.474860	4.789750	12.045263
Au	11.636347	5.313459	13.606502
Au	14.338530	5.985752	13.566461
Au	7.142282	7.175614	12.080679
Au	9.947372	7.551203	12.505114
Ir	12.413646	7.969175	11.022744
Au	15.041800	8.666870	13.637621
Au	7.715904	9.280609	13.700720
Au	10.418984	9.937848	14.018251
Au	12.838668	10.400070	12.530259
Au	15.621198	10.819105	12.083194
Au	8.443902	11.953650	13.677611
Au	11.132701	12.627566	13.719440
Au	13.254611	13.198254	12.120786
Au	15.962674	13.556326	11.712208

$Ir_{32}Au_{32}$.01

Ir	7.833073	5.183070	5.123658
Ir	10.338469	5.173951	5.106316
Ir	12.911529	5.173962	5.106316
Ir	15.416922	5.183080	5.123659
Ir	7.823951	7.688468	5.106317
Ir	10.316101	7.666102	5.111450
Ir	12.933890	7.666111	5.111450
Ir	15.426046	7.688473	5.106315
Ir	7.823961	10.261528	5.106316
Ir	10.316111	10.283891	5.111452

Ir	12.933899	10.283901	5.111445
Ir	15.426052	10.261535	5.106317
Ir	7.833078	12.766925	5.123659
Ir	10.338474	12.776045	5.106316
Ir	12.911535	12.776051	5.106317
Ir	15.416928	12.766927	5.123666
Ir	7.838881	5.188879	7.590935
Ir	10.336358	5.159916	7.559031
Ir	12.913642	5.159923	7.559028
Ir	15.411116	5.1888840	7.590930
Ir	7.809914	7.686356	7.559030
Ir	10.311494	7.661494	7.548137
Ir	12.938502	7.661498	7.548136
Ir	15.440080	7.686357	7.559029
Ir	7.809924	10.263639	7.559030
Ir	10.311499	10.288502	7.548136
Ir	12.938505	10.288505	7.548137
Ir	15.440084	10.263644	7.559030
Ir	7.838884	12.761116	7.590935
Ir	10.336360	12.790079	7.559031
Ir	12.913645	12.790084	7.559029
Ir	15.411117	12.761118	7.590935
Au	7.520937	4.870937	10.190355
Au	10.284724	4.673115	10.196374
Au	12.965281	4.673115	10.196371
Au	15.729066	4.870935	10.190349
Au	7.323114	7.634724	10.196373
Au	10.237426	7.587426	10.306803
Au	13.012565	7.587427	10.306803
Au	15.926871	7.634723	10.196374
Au	7.323115	10.315281	10.196373
Au	10.237427	10.362564	10.306804
Au	13.012566	10.362566	10.306802
Au	15.926871	10.315280	10.196375
Au	7.520934	13.079067	10.190352
Au	10.284722	13.276868	10.196375
Au	12.965279	13.276869	10.196374
Au	15.729066	13.079068	10.190354
Au	7.591312	4.941312	12.928791
Au	10.299116	4.856575	12.955374
Au	12.950883	4.856577	12.955372
Au	15.658686	4.941314	12.928785
Au	7.506573	7.649116	12.955374
Au	10.272858	7.622859	13.165688
Au	12.977141	7.622861	13.165689
Au	15.743428	7.649118	12.955375
Au	7.506575	10.300884	12.955375
Au	10.272860	10.327142	13.165689
Au	12.977143	10.327143	13.165686
Au	15.743430	10.300886	12.955375
Au	7.591313	13.008687	12.928788
Au	10.299117	13.093431	12.955376
Au	12.950884	13.093432	12.955375
Au	15.658687	13.008689	12.928790

$Ir_{32}Au_{32}$.02

Ir	8.057795	6.123896	3.753625
Ir	10.158451	5.008287	4.737695

Ir	12.431552	5.004111	5.746544
Ir	14.195341	5.801401	4.207409
Ir	7.507382	7.974361	5.407364
Ir	10.023591	7.583396	4.907803
Ir	12.517060	7.607936	5.830668
Ir	15.074058	7.713063	5.625492
Ir	7.639674	10.436074	5.124242
Ir	10.313180	9.965541	4.573603
Ir	12.587471	10.108059	5.659822
Ir	15.163135	10.125479	5.557307
Ir	8.837712	11.573190	3.209321
Ir	9.682244	12.380715	5.395645
Ir	12.065079	12.676069	6.040934
Ir	13.766653	11.980386	4.373660
Au	7.810415	5.326492	6.372615
Au	10.320993	4.626851	7.476193
Au	12.930095	4.643380	8.422809
Au	15.158674	5.136544	6.700021
Ir	7.965296	7.793060	7.903948
Ir	10.451957	7.741678	7.465353
Ir	12.850799	7.729813	8.490285
Ir	15.339769	7.700850	8.146022
Ir	7.988584	10.254888	7.740621
Ir	10.491743	10.264093	7.294759
Ir	12.880346	10.228124	8.393597
Ir	15.362712	10.186475	8.105524
Au	7.335812	12.742075	6.746446
Au	10.091658	13.657121	7.744679
Au	12.788630	13.256335	8.561686
Au	14.916587	12.694902	6.716890
Au	8.101046	5.252249	9.112595
Au	10.720911	4.769547	10.206452
Au	13.332297	4.785744	11.164308
Au	15.524146	5.194376	9.414132
Ir	8.256679	7.889054	10.381216
Ir	10.751441	7.833175	10.079203
Ir	13.183669	7.828739	11.084981
Ir	15.665421	7.780655	10.667505
Ir	8.294711	10.365839	10.220342
Ir	10.778064	10.314857	9.947328
Ir	13.198116	10.279320	10.998522
Ir	15.690806	10.236675	10.604633
Au	7,.34887	13.025966	9.415128
Au	10.690685	13.278775	10.411498
Au	13.353860	13.225565	11.253729
Au	15.463511	12.782277	9.382538
Au	8.543247	5.377809	11.843832
Au	11.154883	5.140730	12.961425
Au	13.781519	5.191392	13.893905
Au	15.955866	5.403065	12.118467
Au	6.881384	7.512854	12.665602
Au	9.781501	7.624878	12.894272
Au	12.335002	7.625064	13.795174
Au	15.118282	7.658173	13.515483
Au	7.112224	10.265751	12.677243
Au	10.027624	10.301602	12.796484
Au	12.559900	10.331680	13.798323
Au	15.331084	10.316343	13.461443
Au	8.589637	12.564956	12.052793
Au	11.228973	12.824583	13.141707

Au	13.916692	12.788419	13.935086
Au	16.037488	12.585236	12.072072

$Ir_{32}Au_{32}$.03

Ir	7.837583	4.652532	4.742408
Ir	10.248593	5.278168	4.542990
Ir	12.648466	5.429289	5.559523
Ir	15.091021	5.874356	5.530885
Ir	7.171639	7.031075	5.186514
Ir	9.699504	7.605775	5.099079
Ir	12.323566	7.919257	5.474883
Ir	14.910724	8.337873	5.555953
Ir	7.979325	9.491582	5.382629
Ir	10.669418	9.951092	5.454162
Ir	13.226855	10.380703	5.534485
Ir	15.758154	10.803951	5.495814
Ir	7.743631	11.946215	5.522914
Ir	10.219821	12.368466	5.619999
Ir	12.678375	12.838503	5.619816
Ir	15.146709	13.21840	5.349772
Ir	8.095751	4.248438	7.179862
Ir	10.460418	4.878188	7.033597
Ir	12.741527	5.335619	8.110117
Ir	15.196103	5.800183	7.960706
Ir	7.341408	6.643252	7.597801
Ir	9.801480	7.321850	7.599422
Ir	12.386084	7.876439	7.996846
Ir	15.052295	8.338184	8.023897
Ir	7.950879	9.269994	7.842372
Ir	10.642108	9.818620	7.964817
Ir	13.273749	10.363749	8.018422
Ir	15.813525	10.914460	7.931060
Ir	7.699423	11.770060	7.942348
Ir	10.190431	12.325898	8.060274
Ir	12.751512	12.878633	8.079306
Ir	15.254837	13.366338	7.812830
Au	7.611542	4.927601	9.754368
Au	10.752670	4.201823	9.634627
Au	14.032470	4.623982	10.370396
Au	16.788882	4.407346	9.555991
Au	6.857075	7.761952	10.007717
Au	11.052808	6.979751	10.240816
Au	13.482362	8.570885	10.481770
Au	15.884422	6.896154	10.487407
Au	6.8923928	10.587638	10.272957
Au	9.449558	9.235395	10.404159
Au	11.822781	10.853831	10.411118
Au	16.265625	9.633220	10.298512
Au	8.429983	13.023149	10.161794
Au	11.286959	13.655400	10.375621
Au	14.078698	14.197682	10.118916
Au	16.462375	12.415633	10.105685
Au	7.133199	6.071566	12.241866
Au	9.214653	4.203868	11.932350
Au	11.934335	4.942094	12.156670
Au	14.549693	5.844711	12.803914
Au	7.610178	8.781416	12.547595

Au	9.768503	6.784679	12.857087
Au	12.428542	7.614369	13.080856
Au	15.228166	8.546346	12.752613
Au	8.319415	11.459743	12.541025
Au	10.333373	9.480520	13.170599
Au	13.049262	10.330050	13.032200
Au	15.702312	11.271352	12.566201
Au	9.318597	14.295823	12.367599
Au	10.933958	12.168843	12.910785
Au	13.437448	13.070736	12.550170
Au	15.956436	14.091361	12.229440

Au_{64}

Au	7.648816	4.898852	4.898421
Au	10.347348	4.841492	4.841449
Au	13.002934	4.841429	4.841481
Au	15.701535	4.897233	4.898736
Au	7.591421	7.597375	4.841436
Au	10.326522	7.576562	4.640517
Au	13.023704	7.576551	4.640628
Au	15.758922	7.595818	4.840773
Au	7.591333	10.252938	4.841493
Au	10.326481	10.273722	4.640645
Au	13.023616	10.273702	4.641166
Au	15.758612	10.251349	4.839046
Au	7.647304	12.951507	4.898573
Au	10.345783	13.008985	4.840581
Au	13.001273	13.008718	4.839219
Au	15.699398	12.949445	4.912477
Au	7.591396	4.841334	7.597023
Au	10.326363	4.640362	7.576504
Au	13.023435	4.640338	7.576510
Au	15.758370	4.841323	7.597214
Au	7.390467	7.576364	7.576441
Au	10.286248	7.536183	7.536294
Au	13.063689	7.536106	7.536170
Au	15.959387	7.576473	7.575937
Au	7.390384	10.273512	7.576569
Au	10.285997	10.313720	7.536250
Au	13.063426	10.313527	7.536900
Au	15.959294	10.273595	7.573774
Au	7.593494	13.008446	7.597254
Au	10.328440	13.209449	7.575828
Au	13.025687	13.209363	7.573898
Au	15.760365	13.008230	7.612123
Au	7.591421	4.841276	10.252620
Au	10.326492	4.640396	10.273595
Au	13.023761	4.640064	10.273594
Au	15.758444	4.848332	10.252905
Au	7.390568	7.576341	10.273522
Au	10.286290	7.536178	10.313773
Au	13.063626	7.535825	10.313750
Au	15.959364	7.582250	10.273047
Au	7.390324	10.273492	10.273679
Au	10.286063	10.313571	10.313808
Au	13.063618	10.313449	10.314255
Au	15.959500	10.279427	10.270733
Au	7.592984	13.008365	10.252922

Au	10.327597	13.209364	10.272917
Au	13.024604	13.209395	10.270888
Au	15.760450	13.015451	10.266179
Au	7.648504	4.898619	12.951168
Au	10.347168	4.841628	13.008636
Au	13.002736	4.841867	13.008587
Au	15.701282	4.897913	12.951904
Au	7.591557	7.597243	13.008612
Au	10.326541	7.576620	13.209627
Au	13.023543	7.576854	13.209856
Au	15.758516	7.596526	13.008147
Au	7.591830	10.252813	13.008660
Au	10.326803	10.273654	13.209840
Au	13.023906	10.273933	13.210173
Au	15.758944	10.252190	13.006254
Au	7.648616	12.951394	12.951603
Au	10.347228	13.008599	13.007881
Au	13.002934	13.008993	13.006487
Au	15.701976	12.951191	12.965386
